# Supplementary material for: An analysis of tissue-specific alternative splicing at the protein level
Source: PLoS Comput Biol. 2020 Oct 5;16(10):e1008287. doi: 10.1371/journal.pcbi.1008287 (PMC7561204; doi:10.1371/journal.pcbi.1008287)
Supplement: S1 Fig — The count of the number of times we recorded tissue specific differences at the protein level in each of the 30 tissues. (PDF) [file pcbi.1008287.s001.pdf]

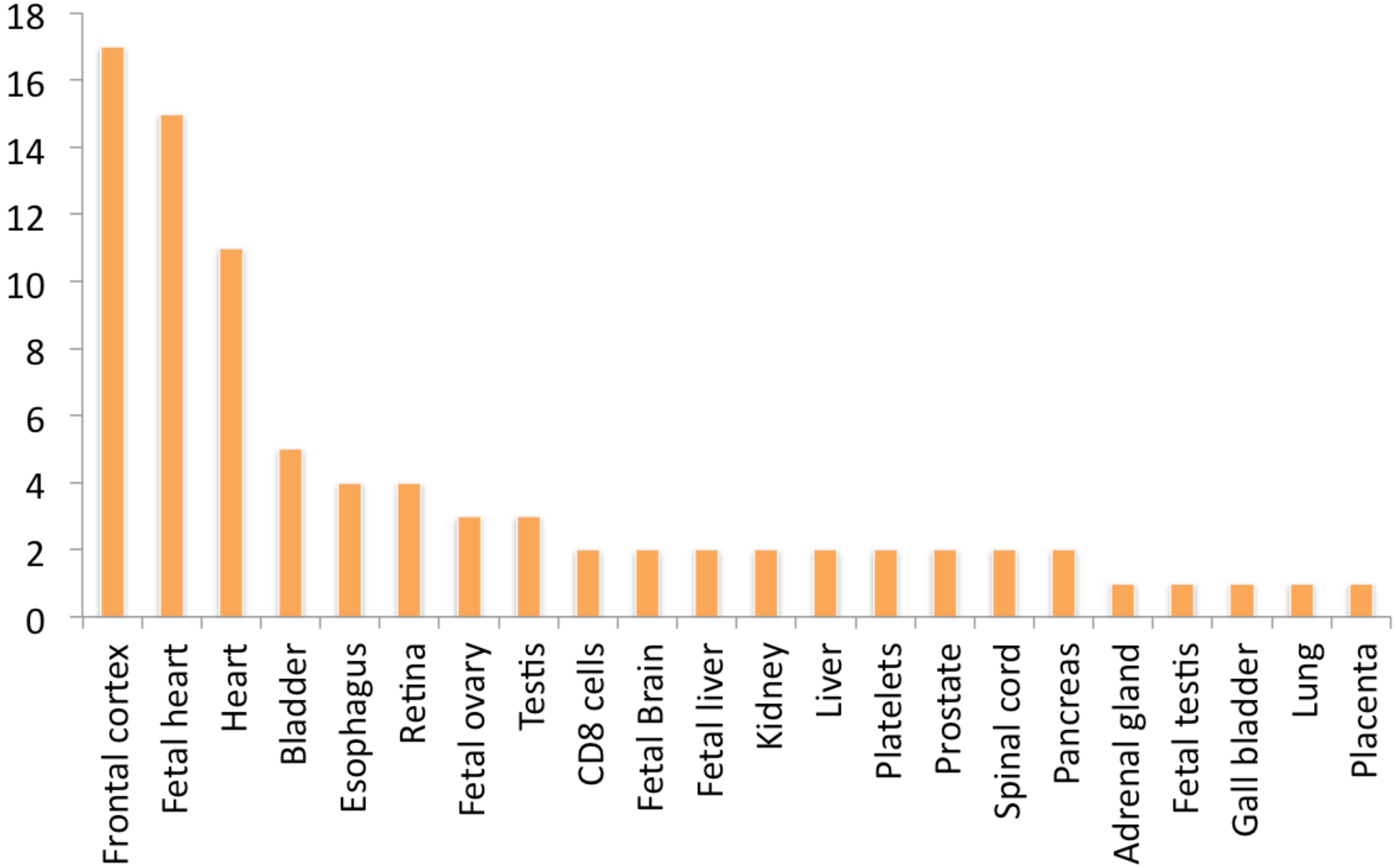


**S1 Figure. Significant tissue specific alternative splicing cases in proteomics tissues.** The count of the number of times we recorded tissue specific differences at the protein level in each of the 30 tissues.
