# Supplementary material for: An analysis of tissue-specific alternative splicing at the protein level
Source: PLoS Comput Biol. 2020 Oct 5;16(10):e1008287. doi: 10.1371/journal.pcbi.1008287 (PMC7561204; doi:10.1371/journal.pcbi.1008287)
Supplement: S2 Fig — Mean order and disorder predicted by IUPred for various subsets. Protein TS are those events that are tissue specific at the protein level. Transcript TS are those events that are tissue specific at the transcript level. Cassette TS are skipped exon events that are tissue-specific at the protein level. Protein Not are those events that are not tissue specific at the protein level. Transcript Not are those events that are not tissue specific at the transcript level. Cassette TS are skipped exon events that are not tissue specific at the protein level. Ancient are those events that manual curation has shown to evolve more than 400 million years ago. Recent are all other events. (PDF) [file pcbi.1008287.s002.pdf]

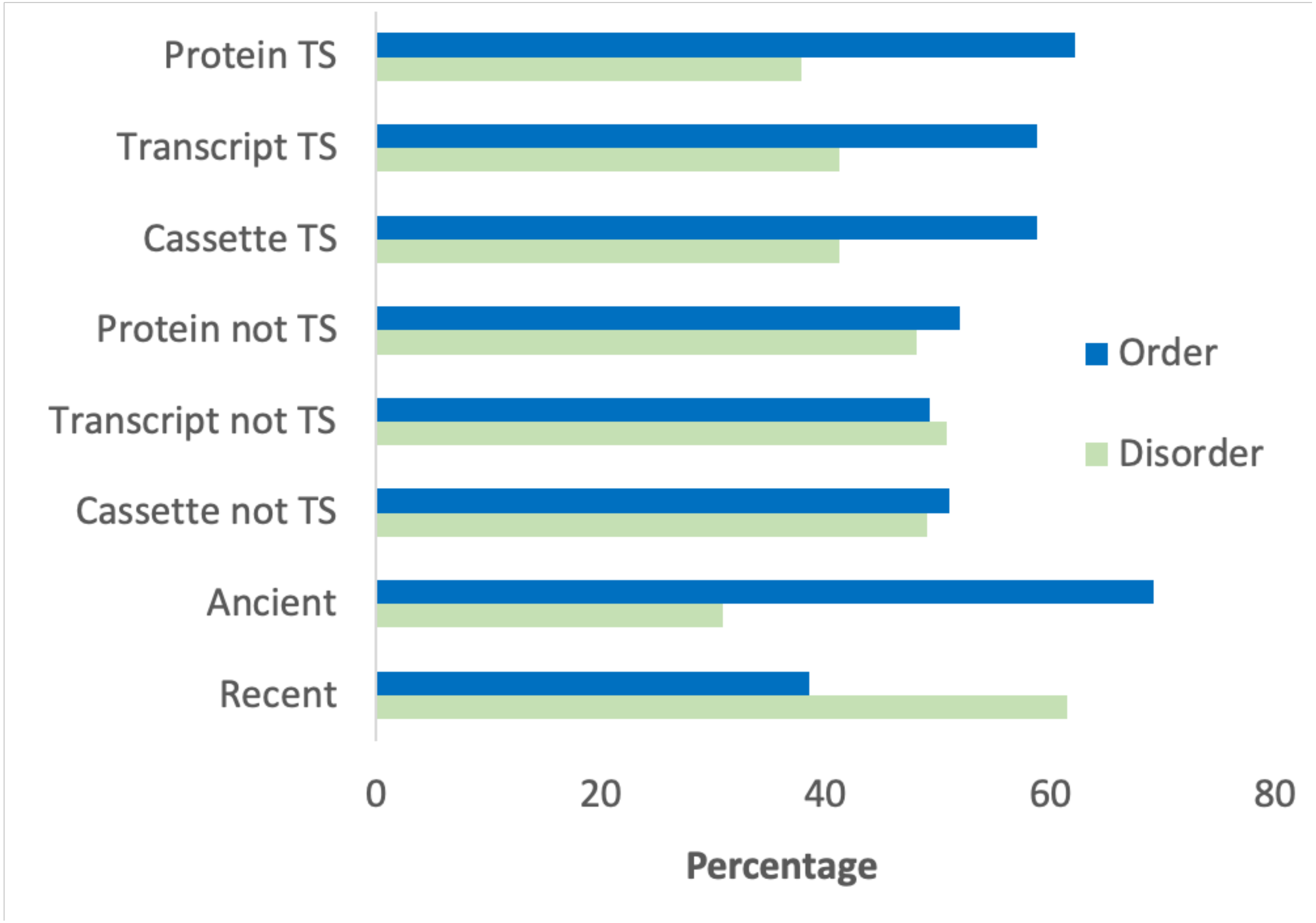

S2 Figure. Predicted order and disorder.

Mean order and disorder predicted by IUPred for various subsets. *Protein TS* are those events that are tissue specific at the protein level. *Transcript TS* are those events that are tissue specific at the transcript level. *Cassette TS* are skipped exon events that are tissue specific at the protein level. *Protein Not* are those events that are not tissue specific at the protein level. *Transcript Not* are those events that are not tissue specific at the transcript level. *Cassette TS* are skipped exon events that are not tissue specific at the protein level. *Ancient* are those events that manual curation has shown to evolve more than 400 million years ago. *Recent* are all other events.
