# Supplementary material for: An analysis of tissue-specific alternative splicing at the protein level
Source: PLoS Comput Biol. 2020 Oct 5;16(10):e1008287. doi: 10.1371/journal.pcbi.1008287 (PMC7561204; doi:10.1371/journal.pcbi.1008287)
Supplement: S3 Fig — The number of events with evidence in four different clades (vertebra to primates) separated into four groups by whether or not they were present in cytoskeleton-related genes (“Cytoskeleton” and “Other genes”), and whether or not the event was found to be significantly tissue specific at the protein level (“TS” or “Not”). There was a significantly higher proportion of vertebrate-derived events among the tissue specific events in cytoskeleton-related genes (Fisher’s exact tests: 0.0093 vs Other genes TS, less than 0.00001 for the other two non-tissue specific groups). (PDF) [file pcbi.1008287.s003.pdf]

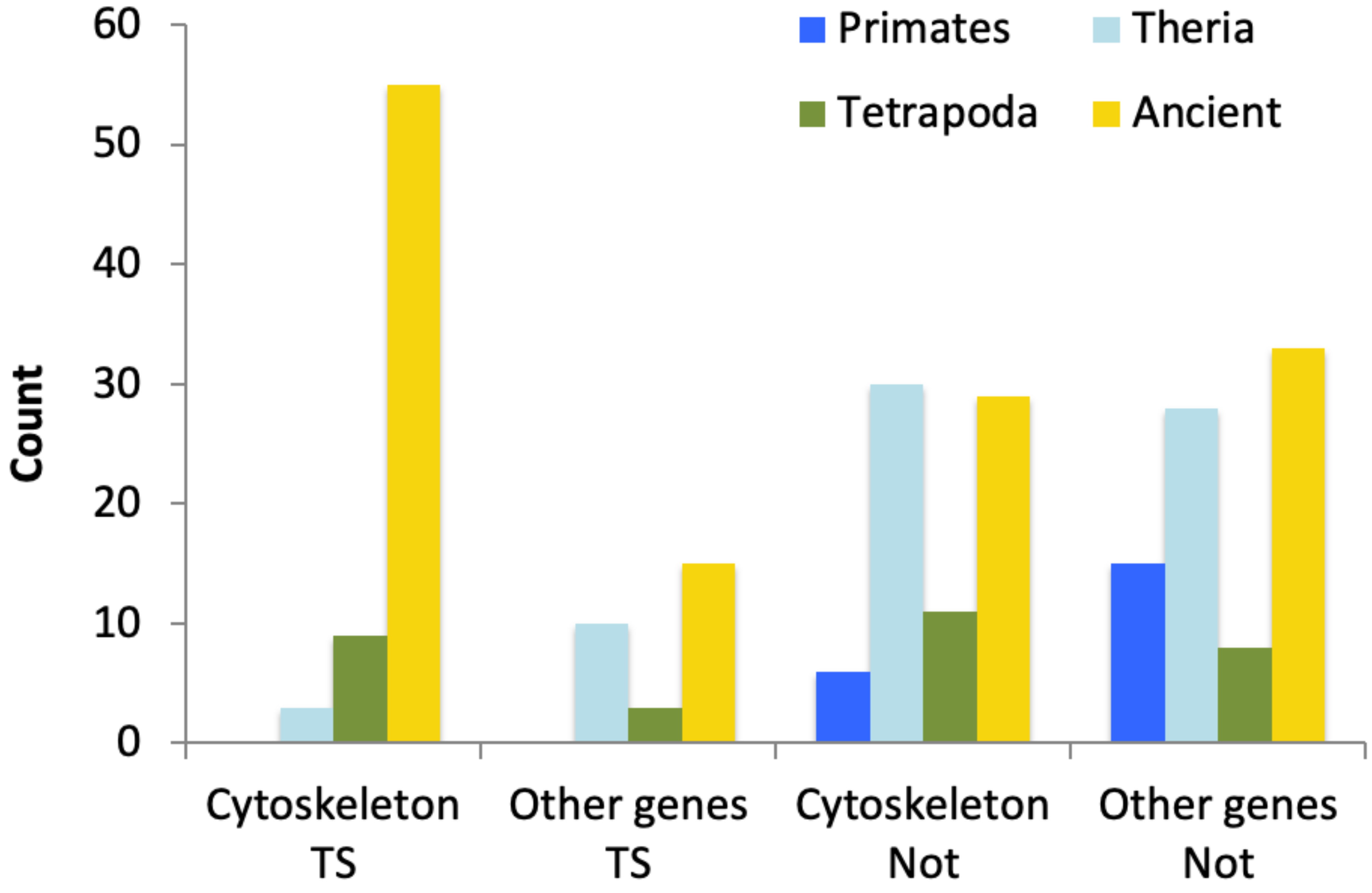

S3 Figure. The relative ages of splice events in cytoskeleton-related genes. The number of events with evidence in four different clades (vertebra to primates) separated into four groups by whether or not they were present in cytoskeleton-related genes ("Cytoskeleton" and "Other genes"), and whether or not the event was found to be significantly tissue specific at the protein level ("TS" or "Not"). There was a significantly higher proportion of vertebrate-derived events among the tissue specific events in cytoskeleton-related genes (Fisher's exact tests: 0.0093 vs Other genes TS).
