# Supplementary figures and images for: An analysis of tissue-specific alternative splicing at the protein level
Source: PLoS Comput Biol. 2020 Oct 5;16(10):e1008287. doi: 10.1371/journal.pcbi.1008287 (PMC7561204; doi:10.1371/journal.pcbi.1008287)

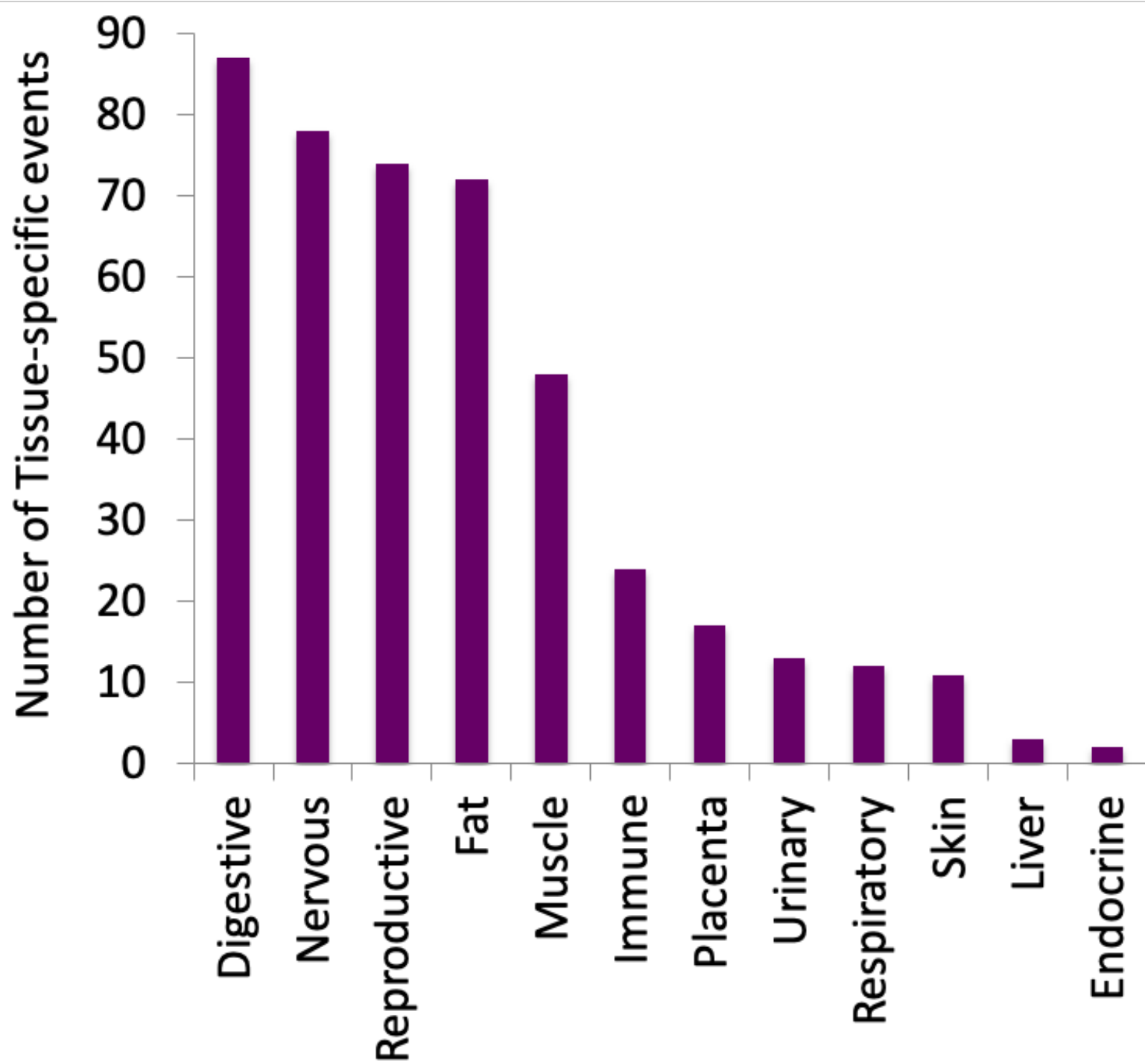

S4 Figure. The number of tissue-specific events in each of the 12 transcriptomics tissue groups

Supplement: S4 Fig — (PDF) [file pcbi.1008287.s004.pdf]
