# Supplementary material for: An analysis of tissue-specific alternative splicing at the protein level
Source: PLoS Comput Biol. 2020 Oct 5;16(10):e1008287. doi: 10.1371/journal.pcbi.1008287 (PMC7561204; doi:10.1371/journal.pcbi.1008287)
Supplement: S5 Fig — For each enriched/depleted event in the corresponding tissue the chart shows the percentage of reads support one side of the event that are detected in the corresponding tissue, plotted against the percentage of all PEDs for the same side of the event detected in proteomics experiments for that tissue. Results are shown just for those events that are enriched/depleted in transcriptomics experiments in (A) digestive, (B) muscle, (C) nervous and (D) reproductive tissues. (PDF) [file pcbi.1008287.s005.pdf]

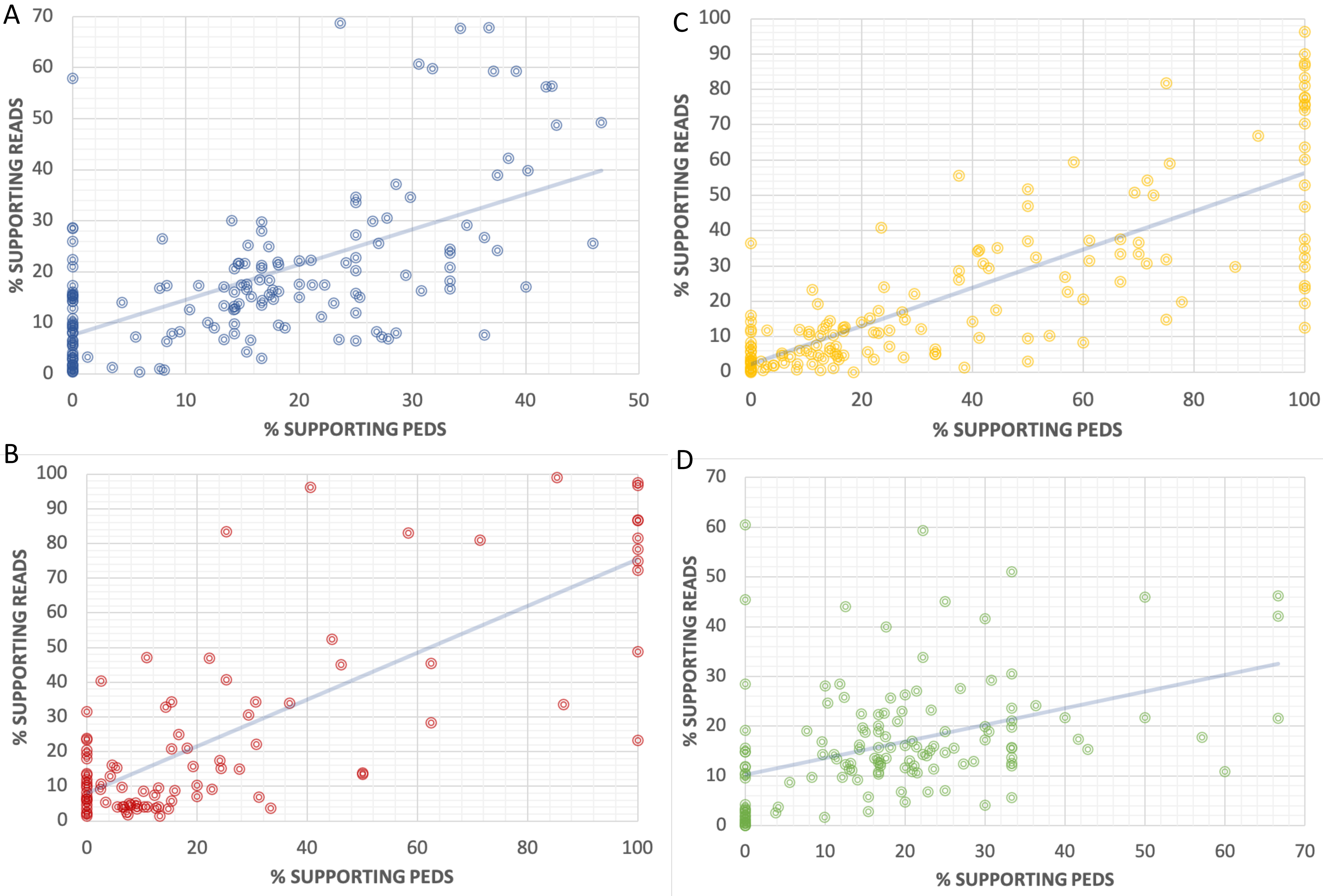

S5 Figure. Correlation between supporting PEDs and supporting reads.
For each enriched/depleted event in the corresponding tissue the chart shows the percentage of reads support one side of the event that are detected in the corresponding tissue, plotted against the percentage of all PEDs for the same side of the event detected in proteomics experiments for that tissue. Results are shown just for those events that are enriched/depleted in transcriptomics experiments in (A) digestive, (B) muscle, (C) nervous and (D) reproductive tissues.
