# Supplementary material for: An analysis of tissue-specific alternative splicing at the protein level
Source: PLoS Comput Biol. 2020 Oct 5;16(10):e1008287. doi: 10.1371/journal.pcbi.1008287 (PMC7561204; doi:10.1371/journal.pcbi.1008287)
Supplement: S6 Fig — The figure shows the percentage of supporting PEDs for the four tested tissue groups (digestive, muscle, nervous and reproductive) from events are enriched (or depleted) in these groups in transcriptomics experiments. The percentage of supporting PEDs among all PEDs detected are shown for the sides of the events that are enriched in transcriptomics experiments (dark red) and for the sides of the events depleted in transcriptomics experiments (light blue). The percentage of PEDs are shown over all events enriched in transcriptomics experiments (All), over the subsets of events enriched in transcriptomics experiments that evolved after the split from fish (Tetrapoda) and over those that evolved after the split from monotremes (Theria). The number of events enriched in transcriptomics experiments and in each subset is shown in the x-axis. Asterisks above the bars show where the number of PEDs supporting the enriched side of the events were significantly different from the number of PEDs on the depleted sides of the events as would be expected if the events were group specific as a whole. (PDF) [file pcbi.1008287.s006.pdf]

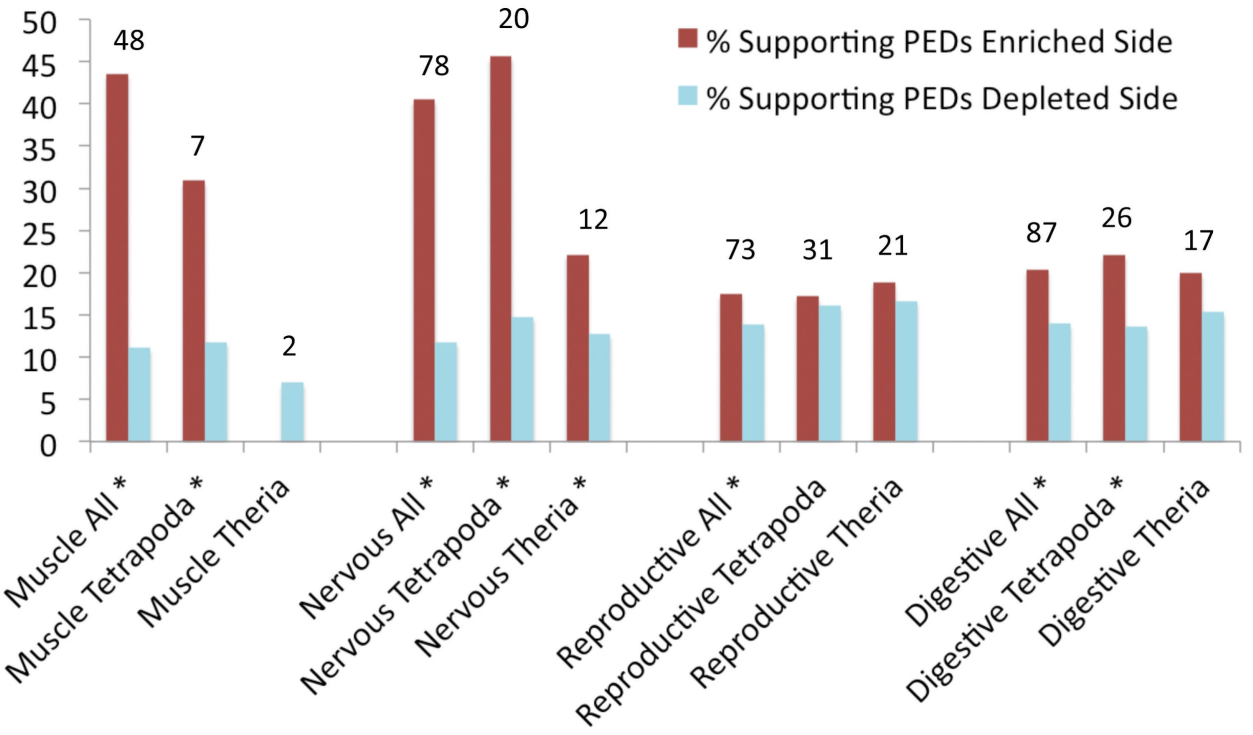

Figure S6. Percentage of PEDs supporting the transcript level enrichment

The figure shows the percentage of supporting PEDs for the four tested tissue groups (digestive, muscle, nervous and reproductive) from events are enriched (or depleted) in these groups in transcriptomics experiments. The percentage of supporting PEDs among all PEDs detected are shown for the sides of the events that are enriched in transcriptomics experiments (dark red) and for the sides of the events depleted in transcriptomics experiments (light blue). The percentage of PEDs are shown over all events enriched in transcriptomics experiments (*All*), over the subsets of events enriched in transcriptomics experiments that evolved after the split from fish (*Tetrapoda*) and over those that evolved after the split from monotremes (*Theria*). The number of events enriched in transcriptomics experiments and in each subset is shown in the x-axis. Asterisks above the bars show where the number of PEDs supporting the enriched side of the events were significantly different from the number of PEDs on the depleted sides of the events as would be expected if the events were group specific as a whole.
