# Supplementary material for: An analysis of tissue-specific alternative splicing at the protein level
Source: PLoS Comput Biol. 2020 Oct 5;16(10):e1008287. doi: 10.1371/journal.pcbi.1008287 (PMC7561204; doi:10.1371/journal.pcbi.1008287)
Supplement: S7 Fig — The darker the colour, the greater the standard deviation. We calculated the mean number of reads across all the tissue groups for each gene (intersect) and used the mean to calculate standard deviations for each set of reads that mapped to each event. Events were counted as tissue group specific when the reads that mapped to the main or alternative (alt) side of the splice event were at least one standard deviation higher than the other side of the splice event. (PDF) [file pcbi.1008287.s007.pdf]

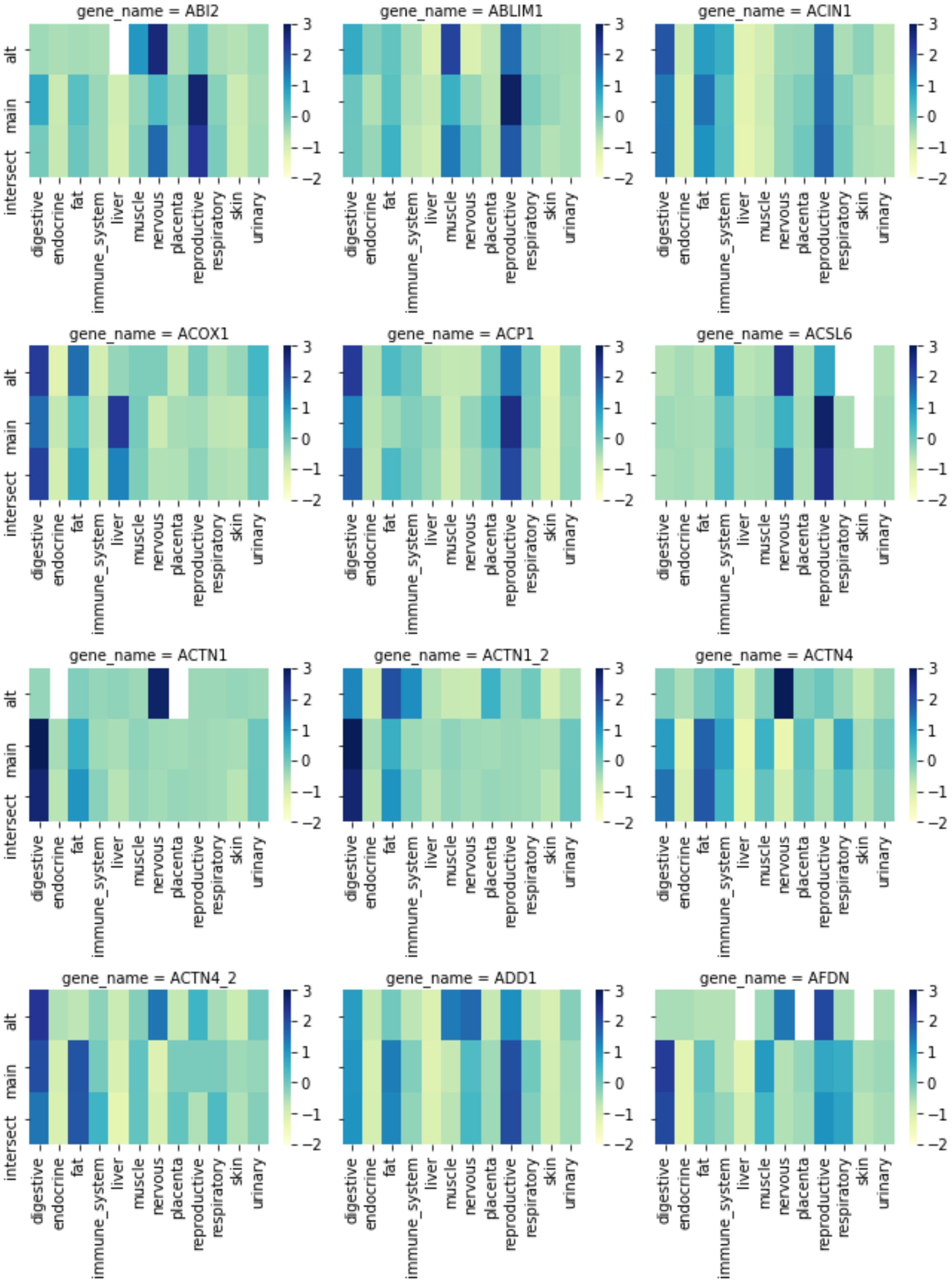
gene_name = ABI2
gene_name = ABLIM1
gene_name = ACIN1
gene_name = ACOX1
gene_name = ACP1
gene_name = ACSL6
gene_name = ACTN1
gene_name = ACTN1_2
gene_name = ACTN4
gene_name = ACTN4_2
gene_name = ADD1
gene_name = AFDN
alt
main
intersect
digestive
endocrine
fat
immune_system
liver
muscle
nervous
placenta
reproductive
respiratory
skin
urinary
3
2
1
0
−1
−2

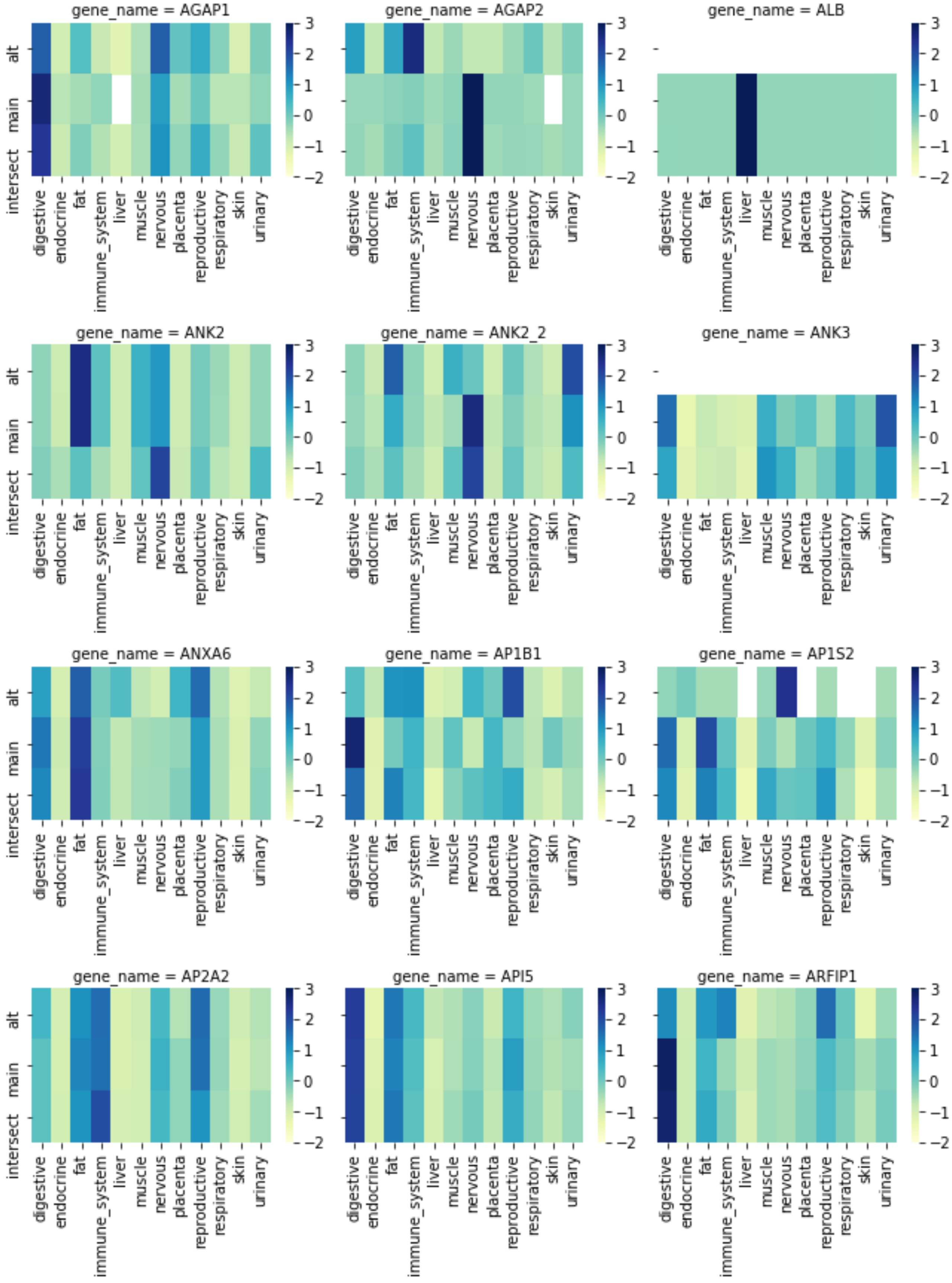

gene_name = AGAP1
gene_name = AGAP2
gene_name = ALB
gene_name = ANK2
gene_name = ANK2_2
gene_name = ANK3
gene_name = ANXA6
gene_name = AP1B1
gene_name = AP1S2
gene_name = AP2A2
gene_name = API5
gene_name = ARFIP1
alt
main
intersect
digestive
endocrine
fat
immune_system
liver
muscle
nervous
placenta
reproductive
respiratory
skin
urinary
3
2
1
0
−1
−2

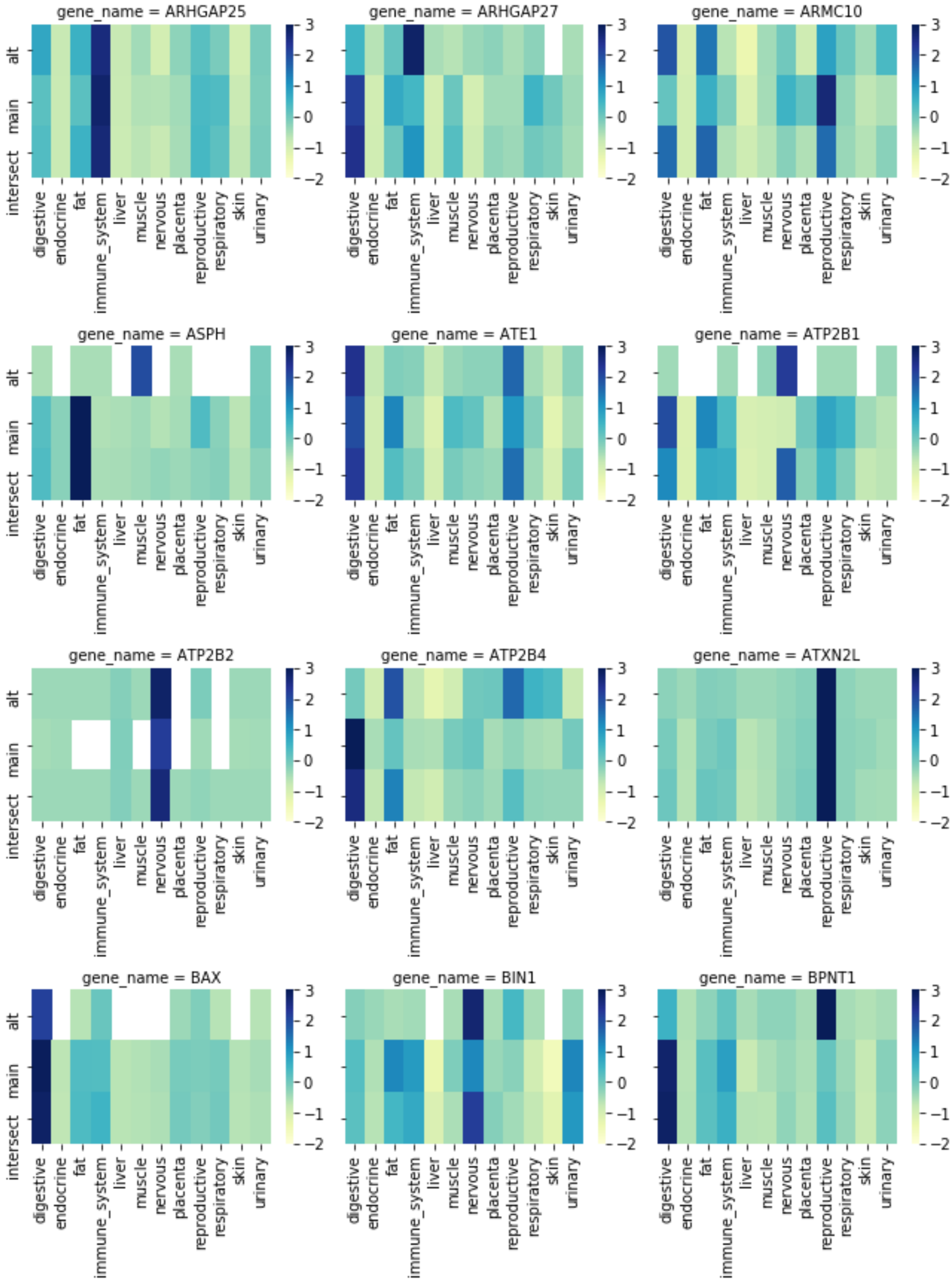

gene_name = ARHGAP25
gene_name = ARHGAP27
gene_name = ARMC10
gene_name = ASPH
gene_name = ATE1
gene_name = ATP2B1
gene_name = ATP2B2
gene_name = ATP2B4
gene_name = ATXN2L
gene_name = BAX
gene_name = BIN1
gene_name = BPNT1
alt
main
intersect
digestive
endocrine
fat
immune_system
liver
muscle
nervous
placenta
reproductive
respiratory
skin
urinary
3
2
1
0
−1
−2

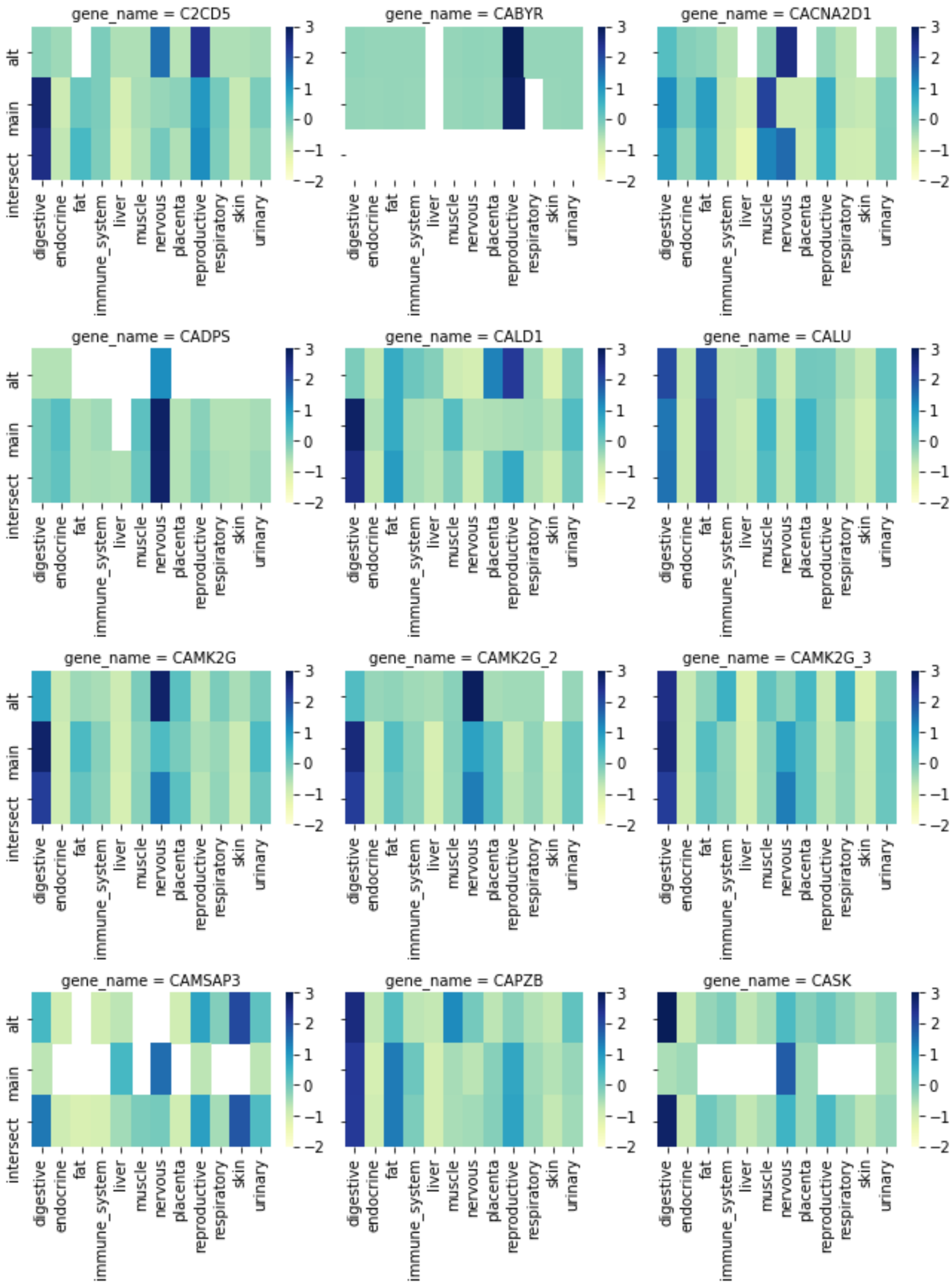
gene_name = C2CD5
gene_name = CABYR
gene_name = CACNA2D1
gene_name = CADPS
gene_name = CALD1
gene_name = CALU
gene_name = CAMK2G
gene_name = CAMK2G_2
gene_name = CAMK2G_3
gene_name = CAMSAP3
gene_name = CAPZB
gene_name = CASK
alt
main
intersect
digestive
endocrine
fat
immune_system
liver
muscle
nervous
placenta
reproductive
respiratory
skin
urinary
3
2
1
0
−1
−2

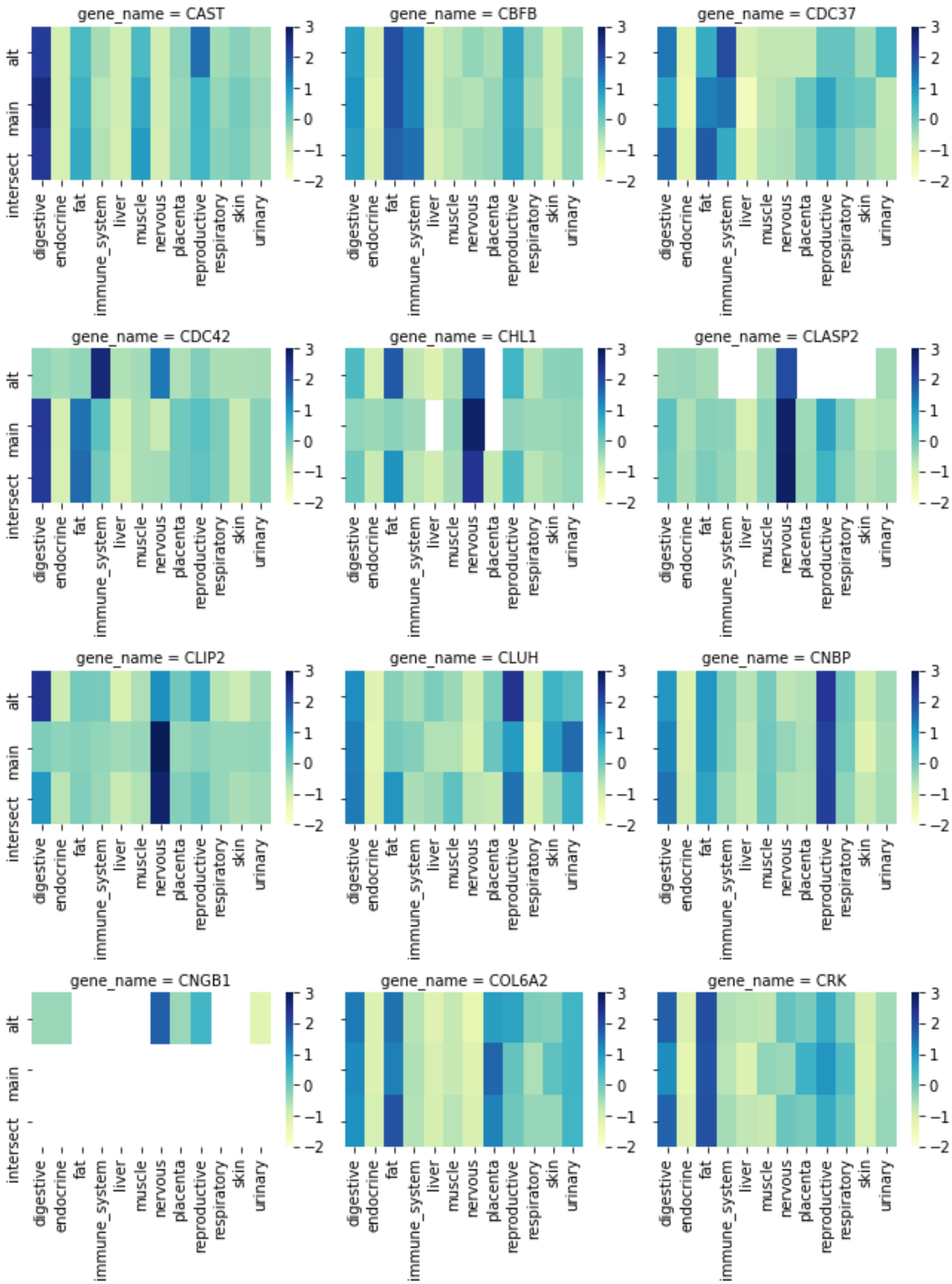

gene_name = CAST
gene_name = CBFB
gene_name = CDC37
gene_name = CDC42
gene_name = CHL1
gene_name = CLASP2
gene_name = CLIP2
gene_name = CLUH
gene_name = CNBP
gene_name = CNGB1
gene_name = COL6A2
gene_name = CRK
alt
main
intersect
digestive
endocrine
fat
immune_system
liver
muscle
nervous
placenta
reproductive
respiratory
skin
urinary
3
2
1
0
−1
−2

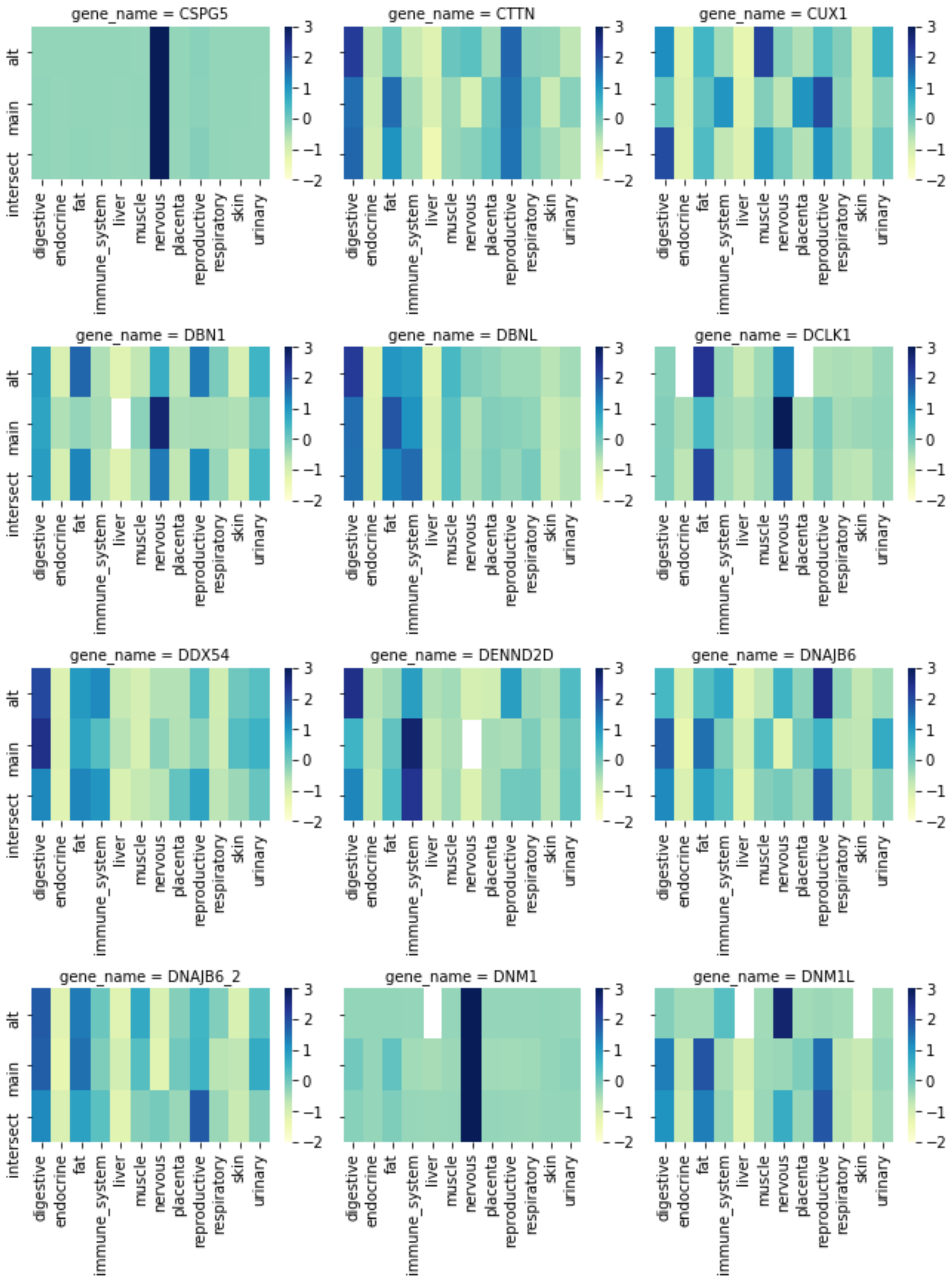
gene_name = CSPG5
gene_name = CTTN
gene_name = CUX1
gene_name = DBN1
gene_name = DBNL
gene_name = DCLK1
gene_name = DDX54
gene_name = DENND2D
gene_name = DNAJB6
gene_name = DNAJB6_2
gene_name = DNM1
gene_name = DNM1L
alt
main
intersect
digestive
endocrine
fat
immune_system
liver
muscle
nervous
placenta
reproductive
respiratory
skin
urinary
3
2
1
0
−1
−2

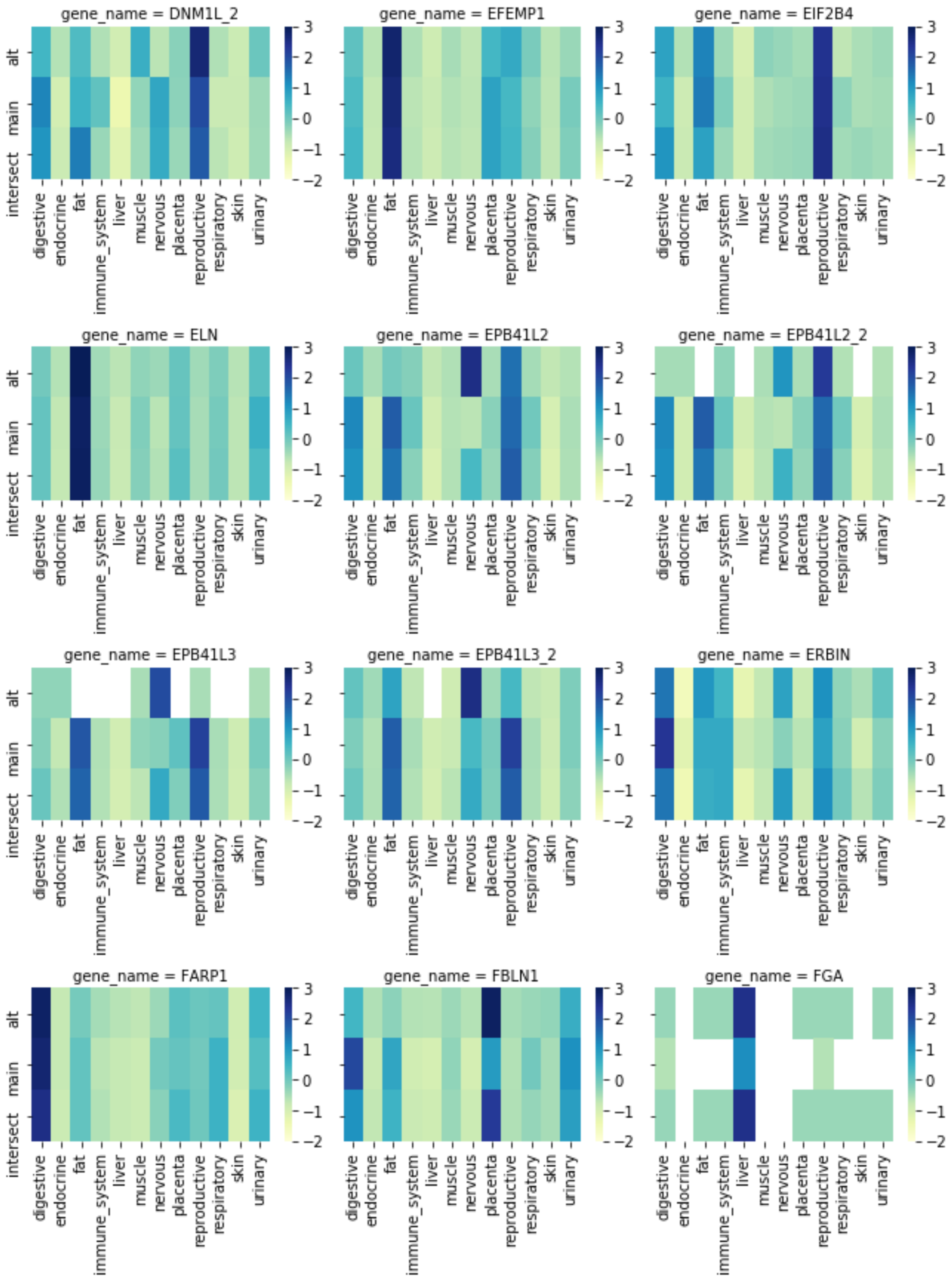
gene_name = DNM1L_2
gene_name = EFEMP1
gene_name = EIF2B4
gene_name = ELN
gene_name = EPB41L2
gene_name = EPB41L2_2
gene_name = EPB41L3
gene_name = EPB41L3_2
gene_name = ERBIN
gene_name = FARP1
gene_name = FBLN1
gene_name = FGA
alt
main
intersect
digestive
endocrine
fat
immune_system
liver
muscle
nervous
placenta
reproductive
respiratory
skin
urinary
3
2
1
0
−1
−2

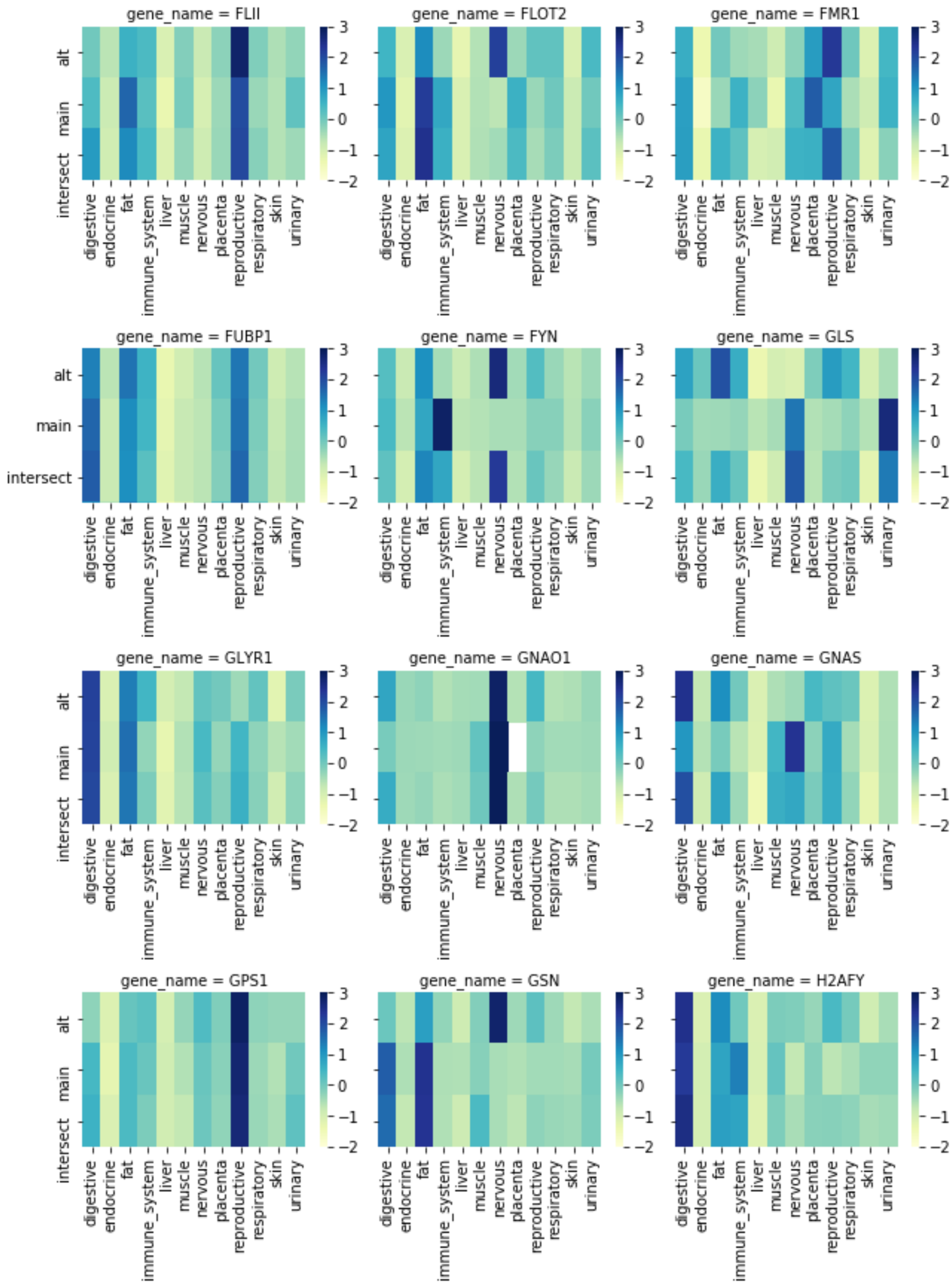
gene_name = FLII
gene_name = FLOT2
gene_name = FMR1
gene_name = FUBP1
gene_name = FYN
gene_name = GLS
gene_name = GLYR1
gene_name = GNAO1
gene_name = GNAS
gene_name = GPS1
gene_name = GSN
gene_name = H2AFY
alt
main
intersect
digestive
endocrine
fat
immune_system
liver
muscle
nervous
placenta
reproductive
respiratory
skin
urinary
3
2
1
0
−1
−2

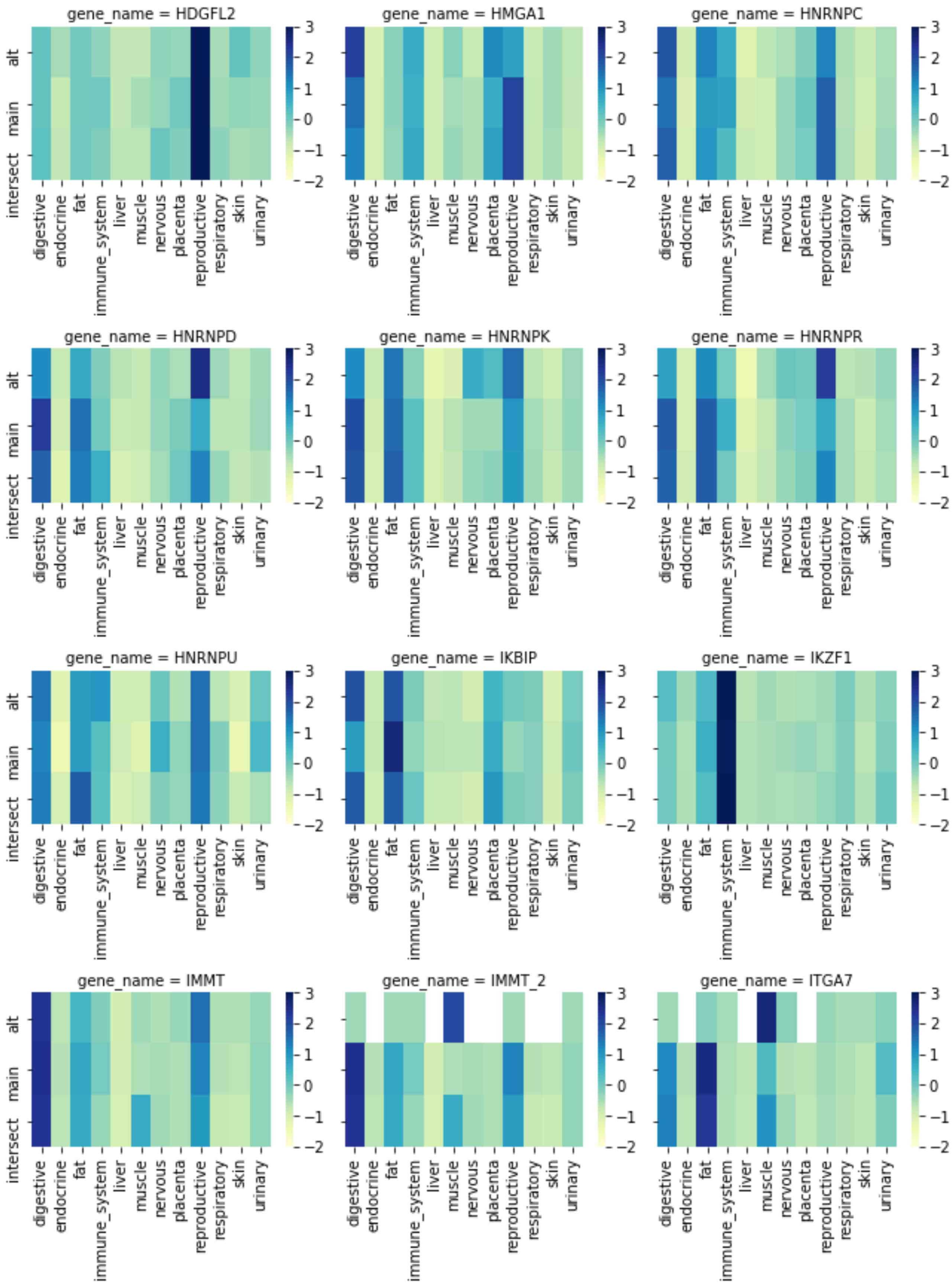
gene_name = HDGFL2
gene_name = HMGA1
gene_name = HNRNPC
gene_name = HNRNPD
gene_name = HNRNPK
gene_name = HNRNPR
gene_name = HNRNPU
gene_name = IKBIP
gene_name = IKZF1
gene_name = IMMT
gene_name = IMMT_2
gene_name = ITGA7
alt
main
intersect
digestive
endocrine
fat
immune_system
liver
muscle
nervous
placenta
reproductive
respiratory
skin
urinary
3
2
1
0
−1
−2

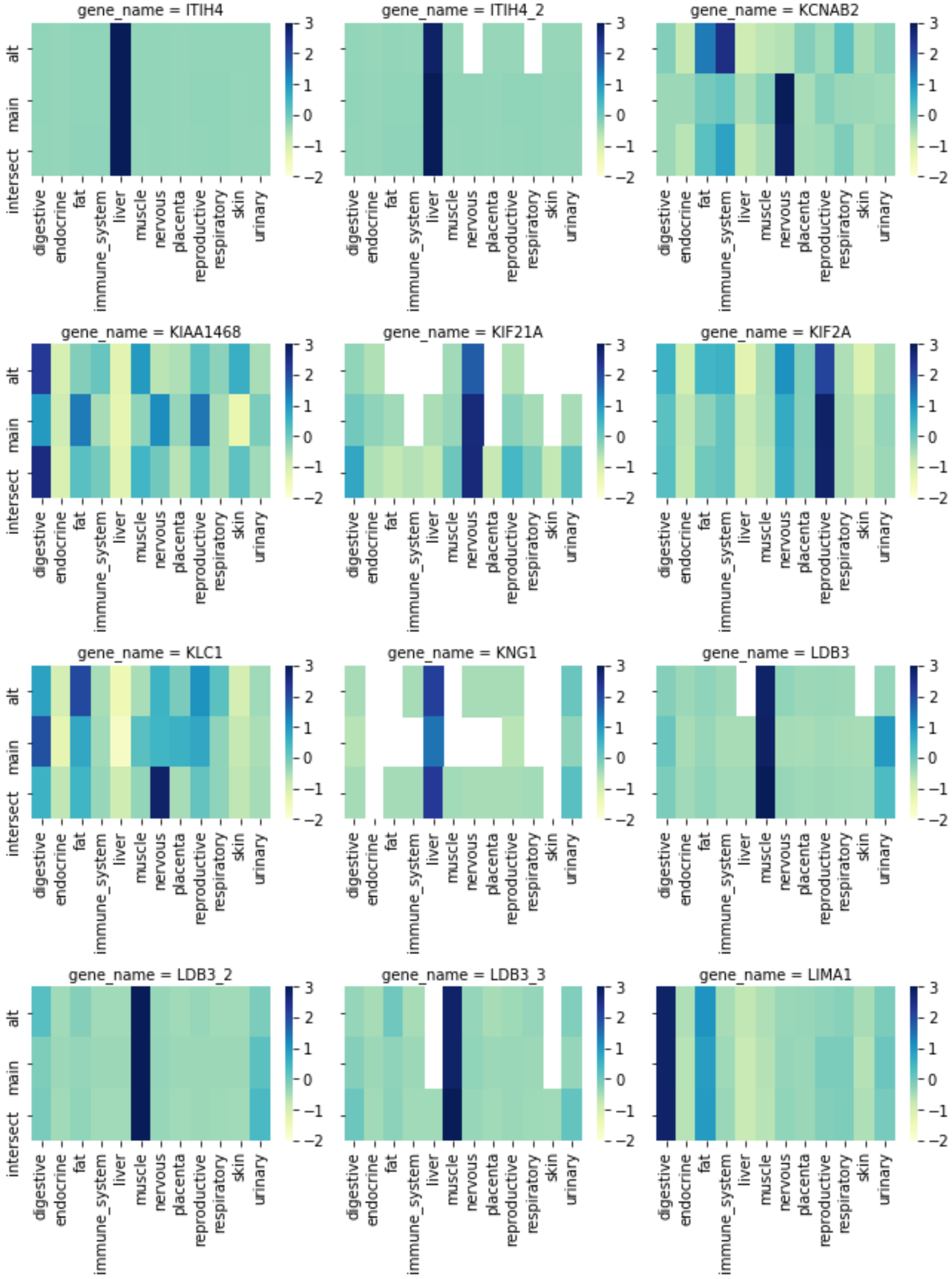
gene_name = ITIH4
gene_name = ITIH4_2
gene_name = KCNAB2
gene_name = KIAA1468
gene_name = KIF21A
gene_name = KIF2A
gene_name = KLC1
gene_name = KNG1
gene_name = LDB3
gene_name = LDB3_2
gene_name = LDB3_3
gene_name = LIMA1
alt
main
intersect
digestive
endocrine
fat
immune_system
liver
muscle
nervous
placenta
reproductive
respiratory
skin
urinary
3
2
1
0
−1
−2

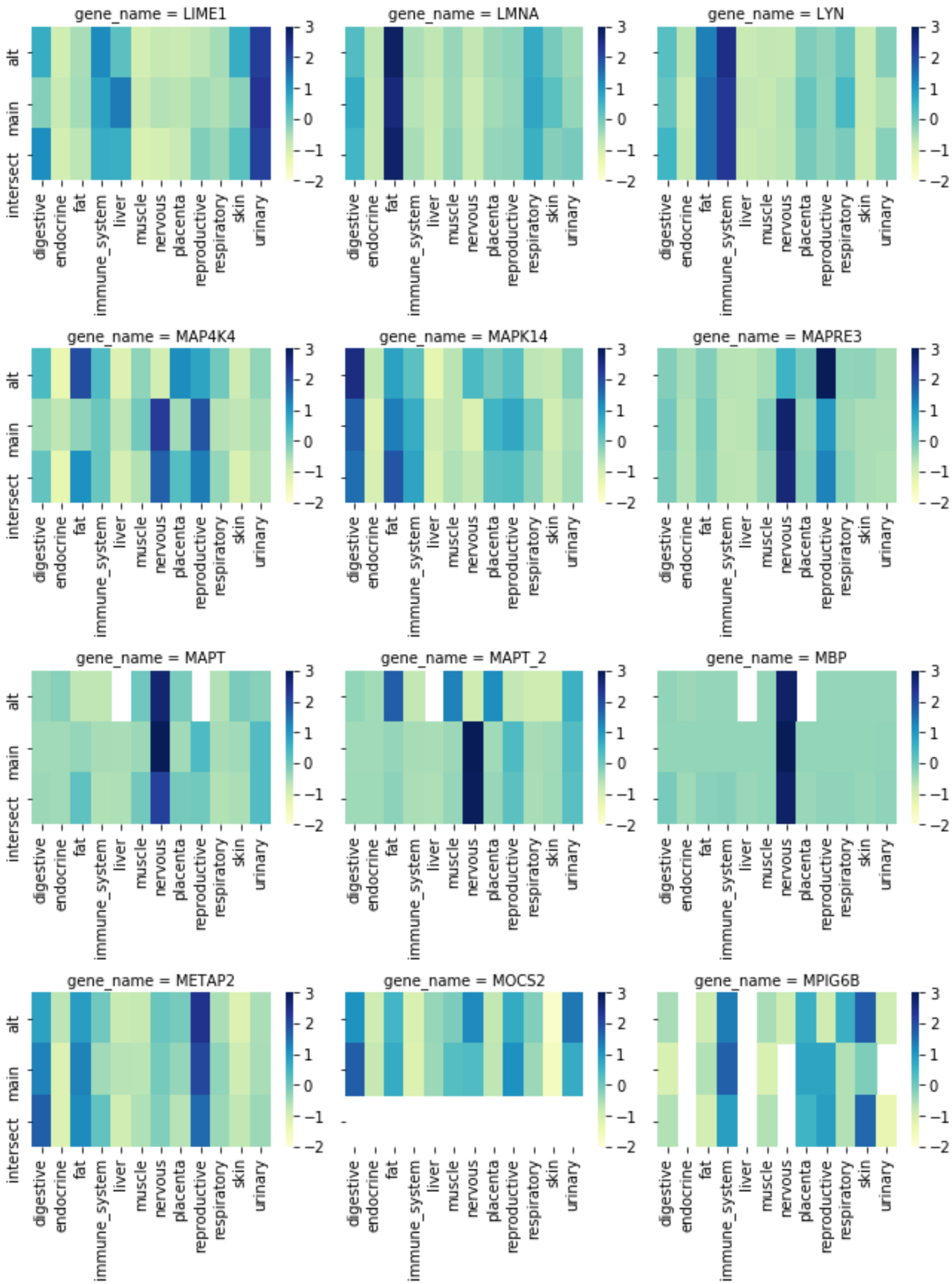
gene_name = LIME1
gene_name = LMNA
gene_name = LYN
gene_name = MAP4K4
gene_name = MAPK14
gene_name = MAPRE3
gene_name = MAPT
gene_name = MAPT_2
gene_name = MBP
gene_name = METAP2
gene_name = MOCS2
gene_name = MPIG6B
alt
main
intersect
digestive
endocrine
fat
immune_system
liver
muscle
nervous
placenta
reproductive
respiratory
skin
urinary
3
2
1
0
−1
−2

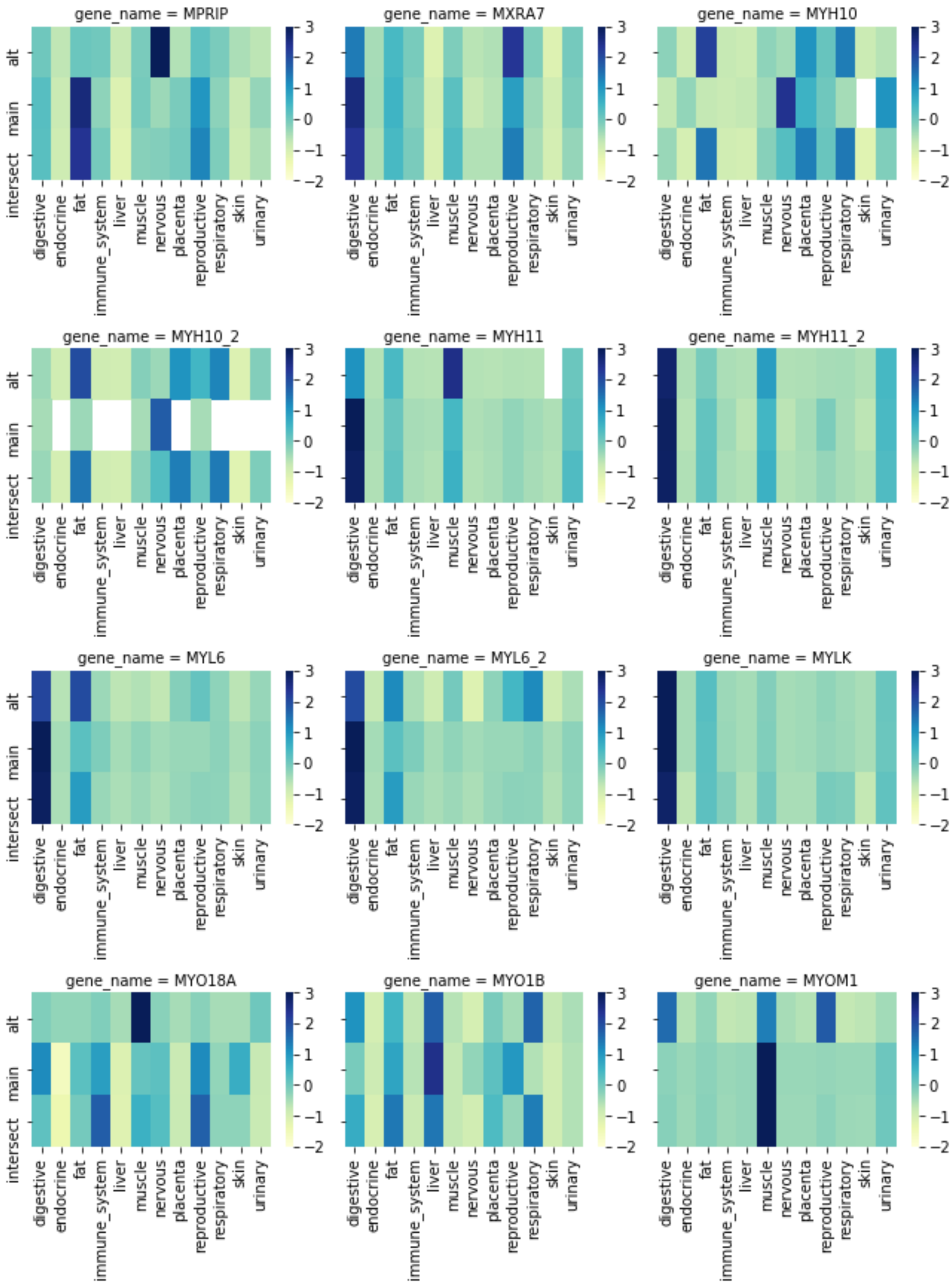

gene_name = MPRIP
gene_name = MXRA7
gene_name = MYH10
gene_name = MYH10_2
gene_name = MYH11
gene_name = MYH11_2
gene_name = MYL6
gene_name = MYL6_2
gene_name = MYLK
gene_name = MYO18A
gene_name = MYO1B
gene_name = MYOM1
alt
main
intersect
digestive
endocrine
fat
immune_system
liver
muscle
nervous
placenta
reproductive
respiratory
skin
urinary
3
2
1
0
−1
−2

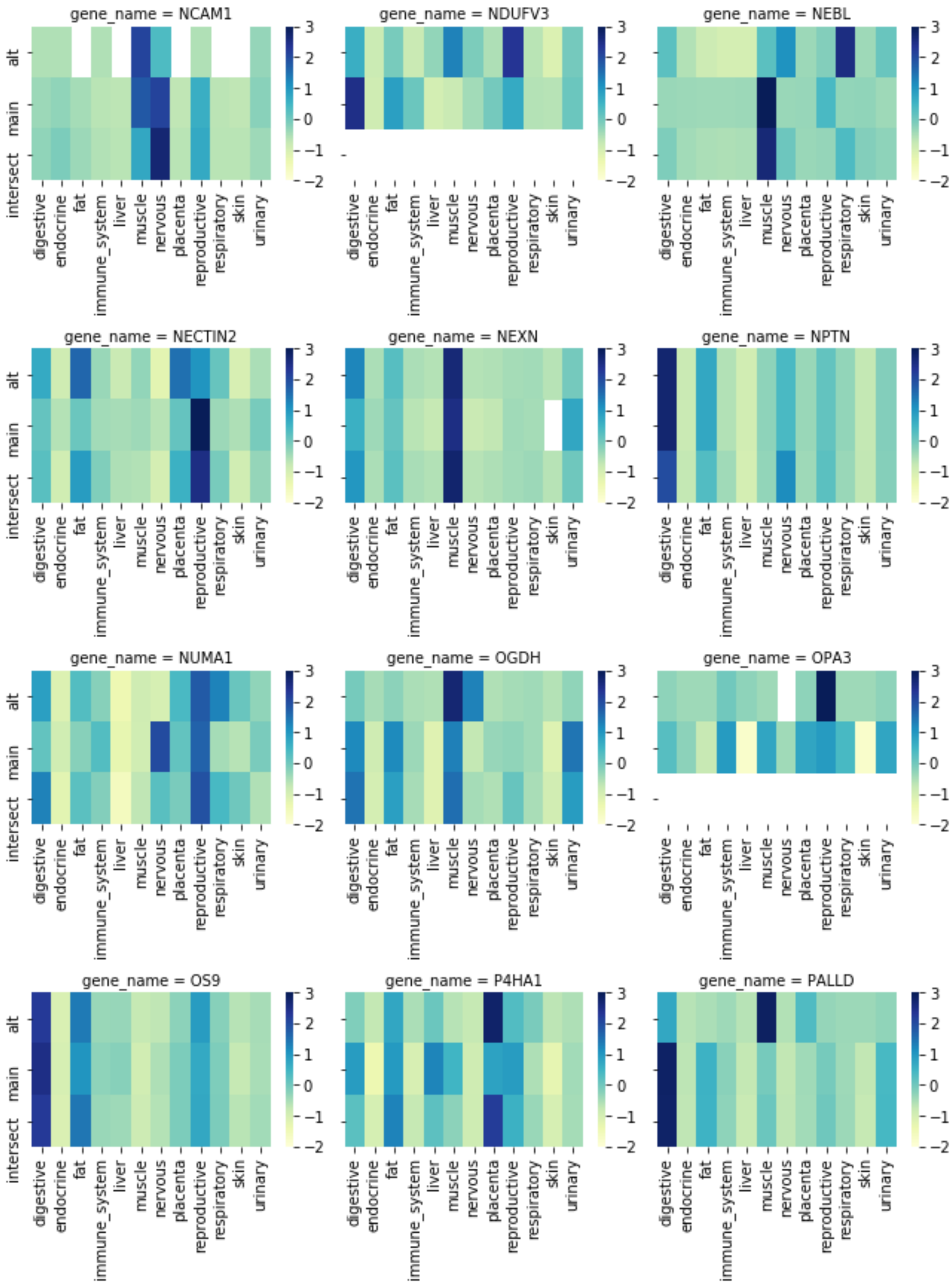
gene_name = NCAM1
gene_name = NDUFV3
gene_name = NEBL
gene_name = NECTIN2
gene_name = NEXN
gene_name = NPTN
gene_name = NUMA1
gene_name = OGDH
gene_name = OPA3
gene_name = OS9
gene_name = P4HA1
gene_name = PALLD
alt
main
intersect
digestive
endocrine
fat
immune_system
liver
muscle
nervous
placenta
reproductive
respiratory
skin
urinary
3
2
1
0
−1
−2

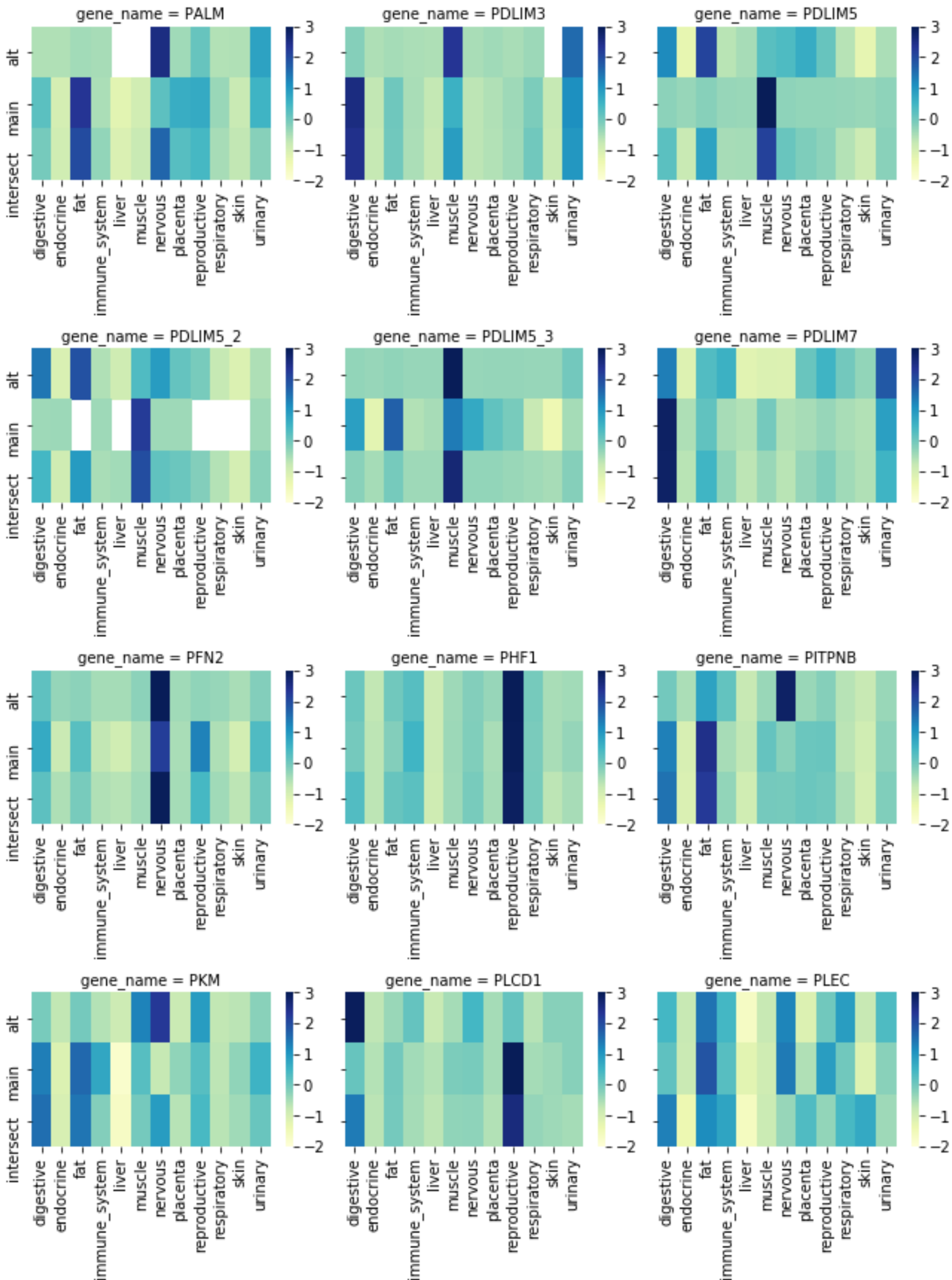
gene_name = PALM
gene_name = PDLIM3
gene_name = PDLIM5
gene_name = PDLIM5_2
gene_name = PDLIM5_3
gene_name = PDLIM7
gene_name = PFN2
gene_name = PHF1
gene_name = PITPNB
gene_name = PKM
gene_name = PLCD1
gene_name = PLEC
alt
main
intersect
digestive
endocrine
fat
immune_system
liver
muscle
nervous
placenta
reproductive
respiratory
skin
urinary
3
2
1
0
−1
−2

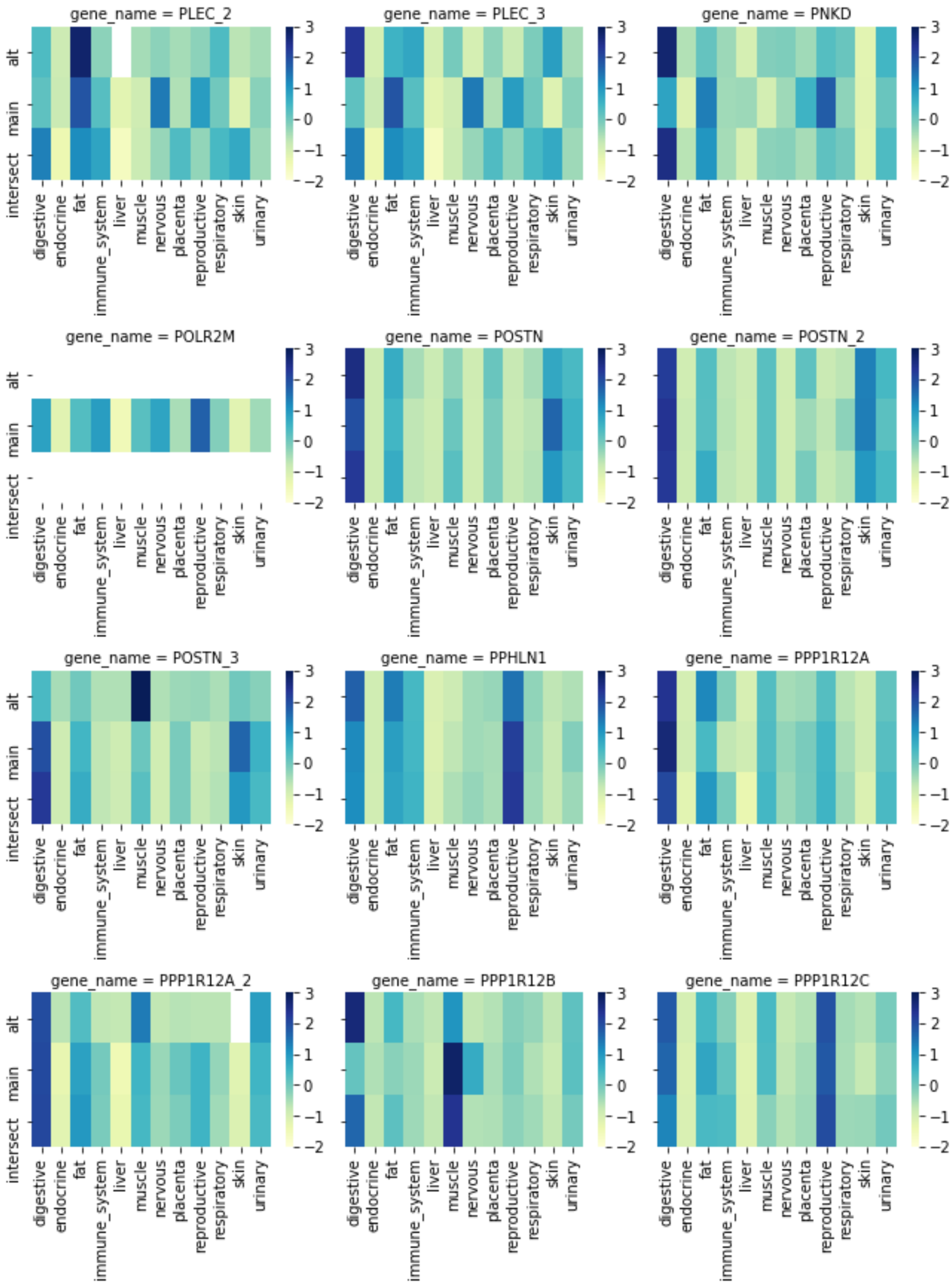
gene_name = PLEC_2
gene_name = PLEC_3
gene_name = PNKD
gene_name = POLR2M
gene_name = POSTN
gene_name = POSTN_2
gene_name = POSTN_3
gene_name = PPHLN1
gene_name = PPP1R12A
gene_name = PPP1R12A_2
gene_name = PPP1R12B
gene_name = PPP1R12C
alt
main
intersect
digestive
endocrine
fat
immune_system
liver
muscle
nervous
placenta
reproductive
respiratory
skin
urinary
3
2
1
0
−1
−2

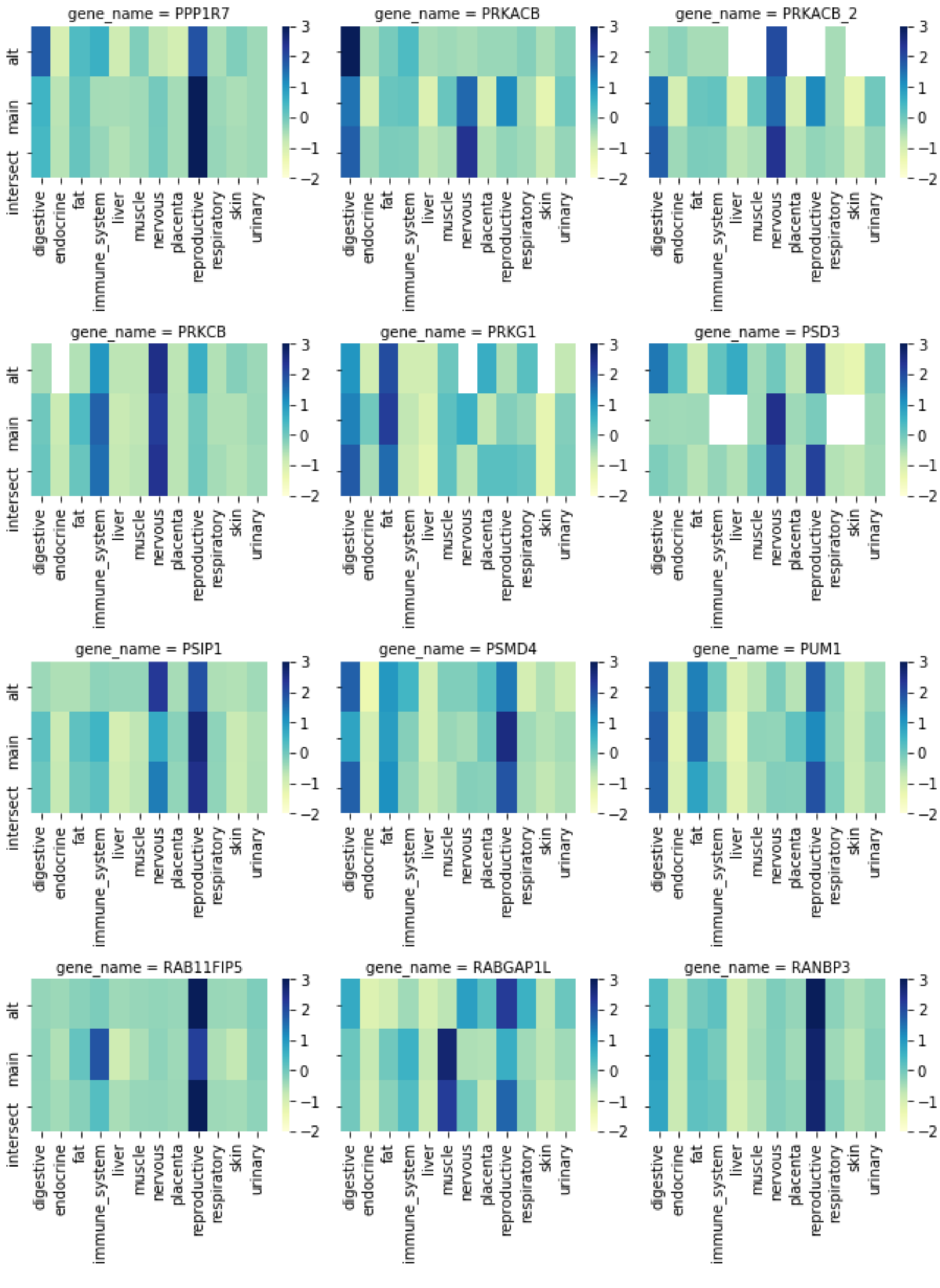
gene_name = PPP1R7
gene_name = PRKACB
gene_name = PRKACB_2
gene_name = PRKCB
gene_name = PRKG1
gene_name = PSD3
gene_name = PSIP1
gene_name = PSMD4
gene_name = PUM1
gene_name = RAB11FIP5
gene_name = RABGAP1L
gene_name = RANBP3
alt
main
intersect
digestive
endocrine
fat
immune_system
liver
muscle
nervous
placenta
reproductive
respiratory
skin
urinary
3
2
1
0
−1
−2

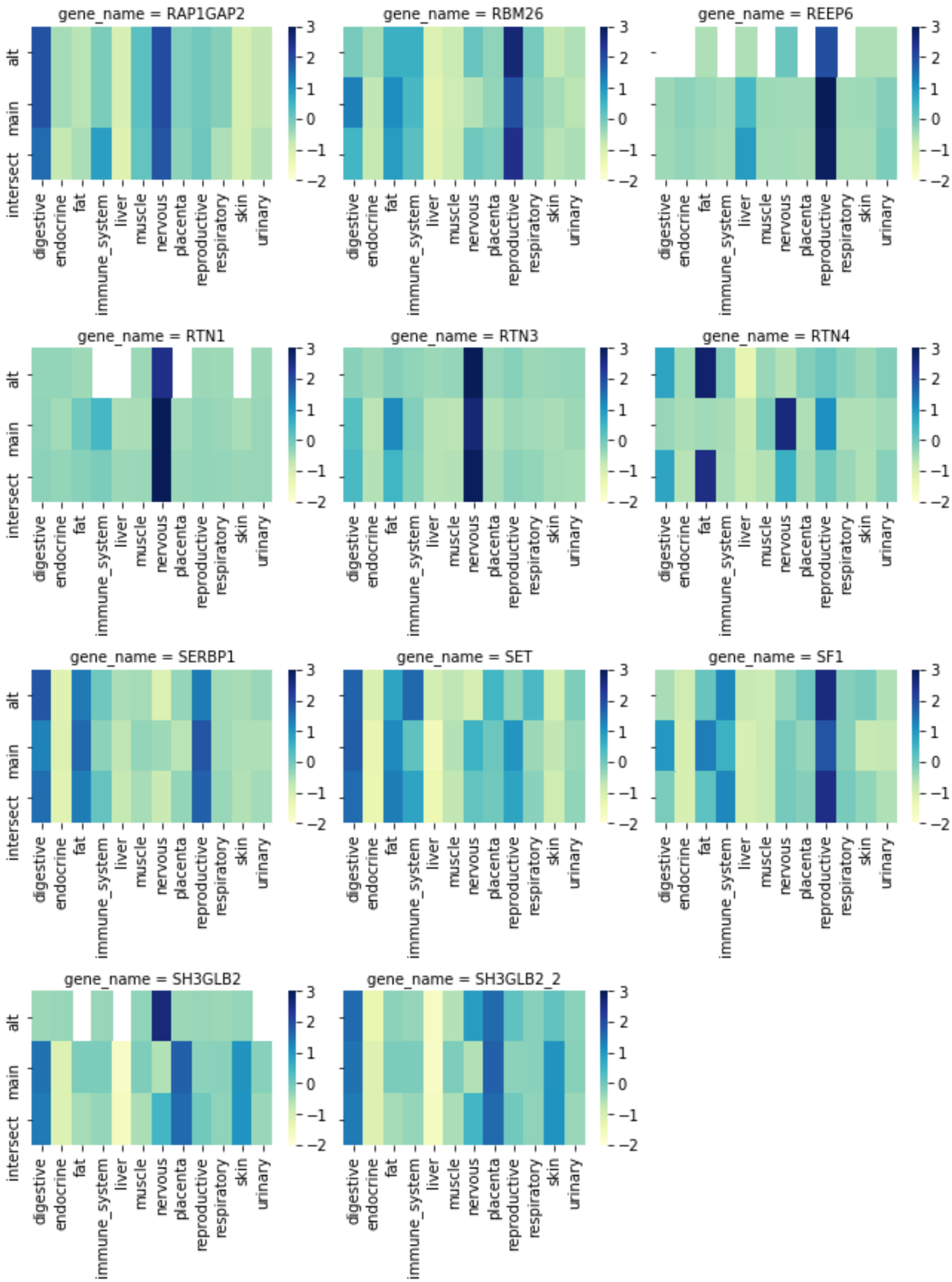
gene_name = RAP1GAP2
gene_name = RBM26
gene_name = REEP6
gene_name = RTN1
gene_name = RTN3
gene_name = RTN4
gene_name = SERBP1
gene_name = SET
gene_name = SF1
gene_name = SH3GLB2
gene_name = SH3GLB2_2
alt
main
intersect
digestive
endocrine
fat
immune_system
liver
muscle
nervous
placenta
reproductive
respiratory
skin
urinary
3
2
1
0
−1
−2

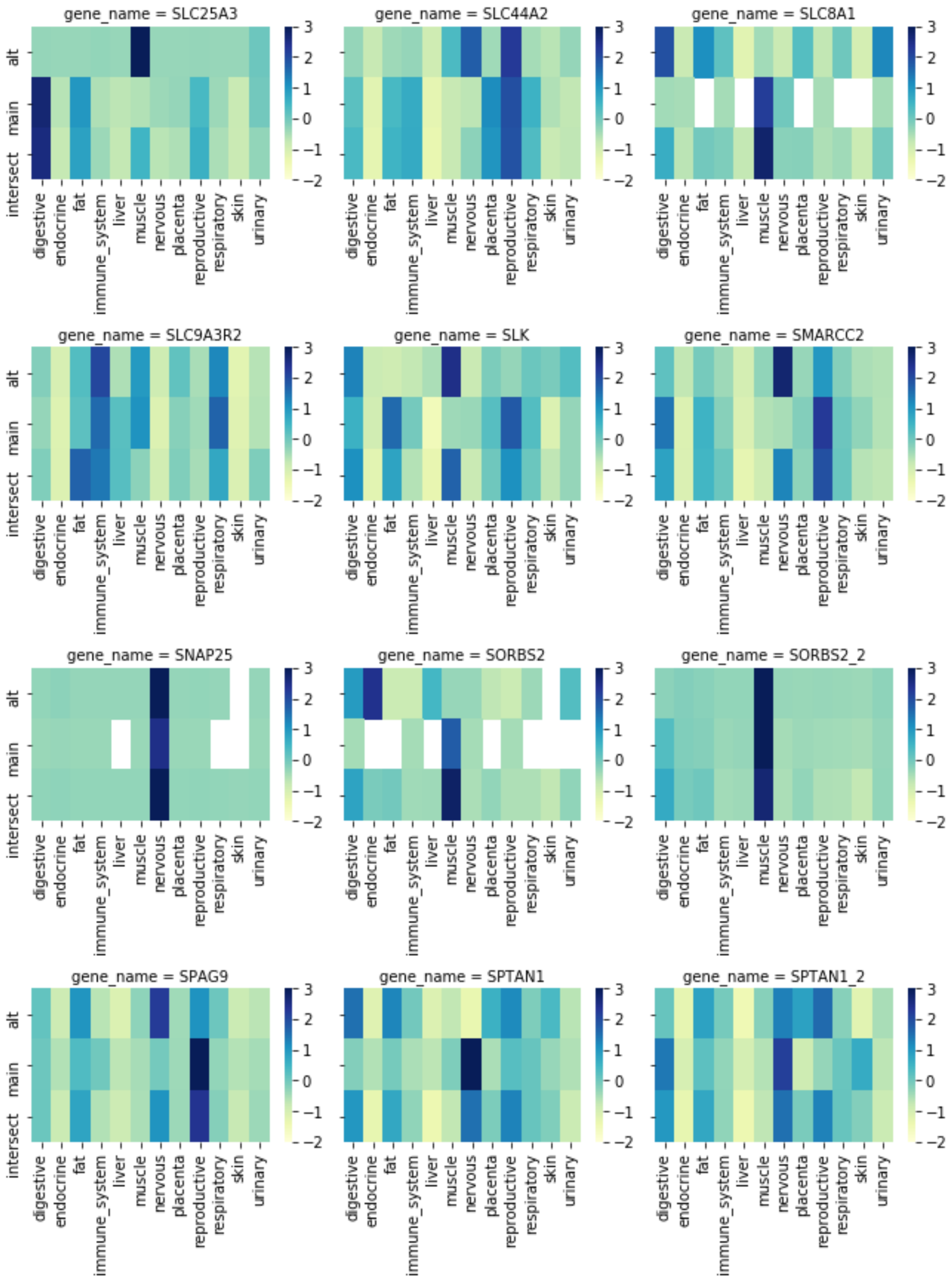
gene_name = SLC25A3
gene_name = SLC44A2
gene_name = SLC8A1
gene_name = SLC9A3R2
gene_name = SLK
gene_name = SMARCC2
gene_name = SNAP25
gene_name = SORBS2
gene_name = SORBS2_2
gene_name = SPAG9
gene_name = SPTAN1
gene_name = SPTAN1_2
alt
main
intersect
digestive
endocrine
fat
immune_system
liver
muscle
nervous
placenta
reproductive
respiratory
skin
urinary
3
2
1
0
−1
−2

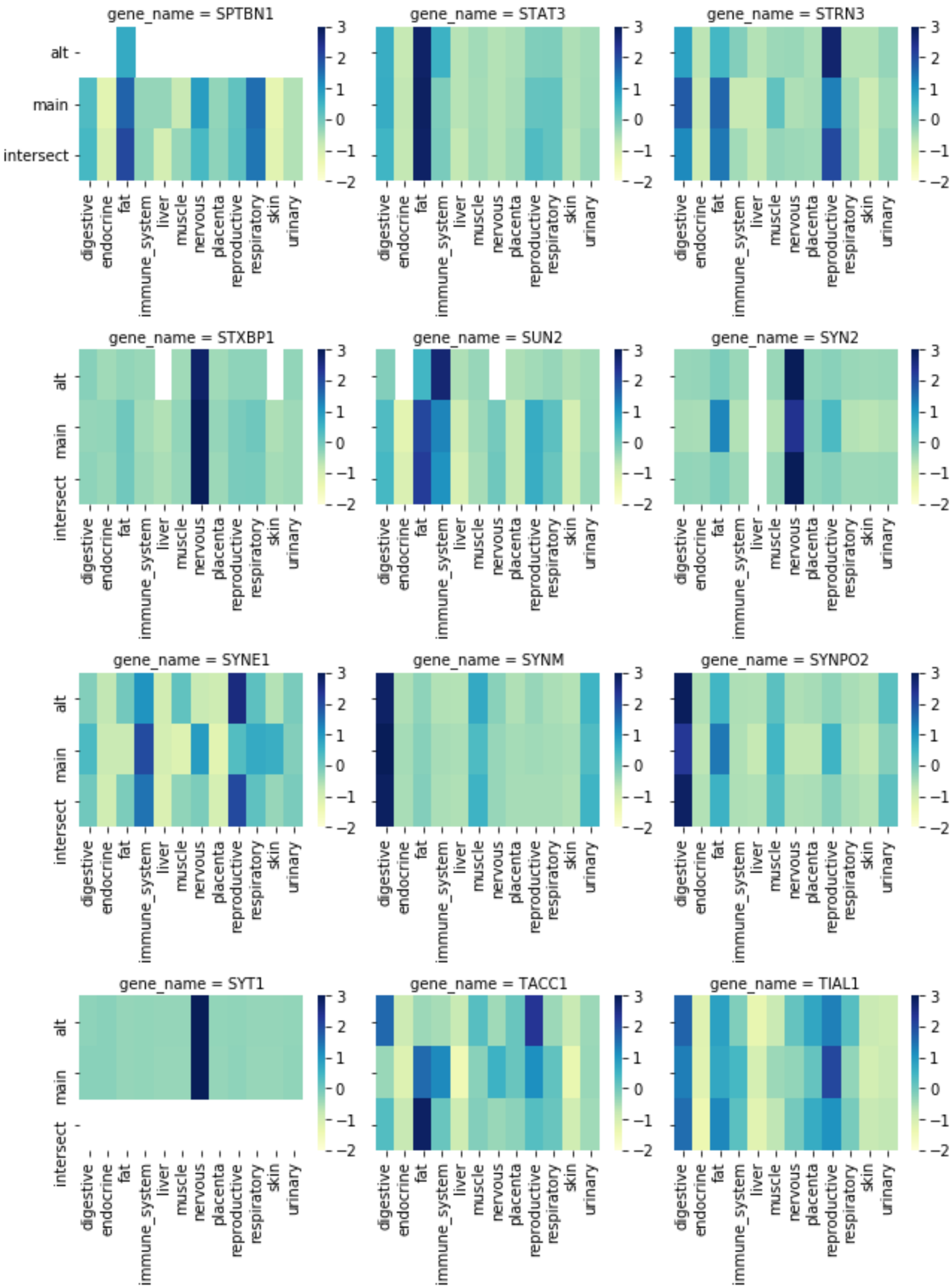
gene_name = SPTBN1
gene_name = STAT3
gene_name = STRN3
gene_name = STXBP1
gene_name = SUN2
gene_name = SYN2
gene_name = SYNE1
gene_name = SYNM
gene_name = SYNPO2
gene_name = SYT1
gene_name = TACC1
gene_name = TIAL1
alt
main
intersect
digestive
endocrine
fat
immune_system
liver
muscle
nervous
placenta
reproductive
respiratory
skin
urinary
3
2
1
0
−1
−2

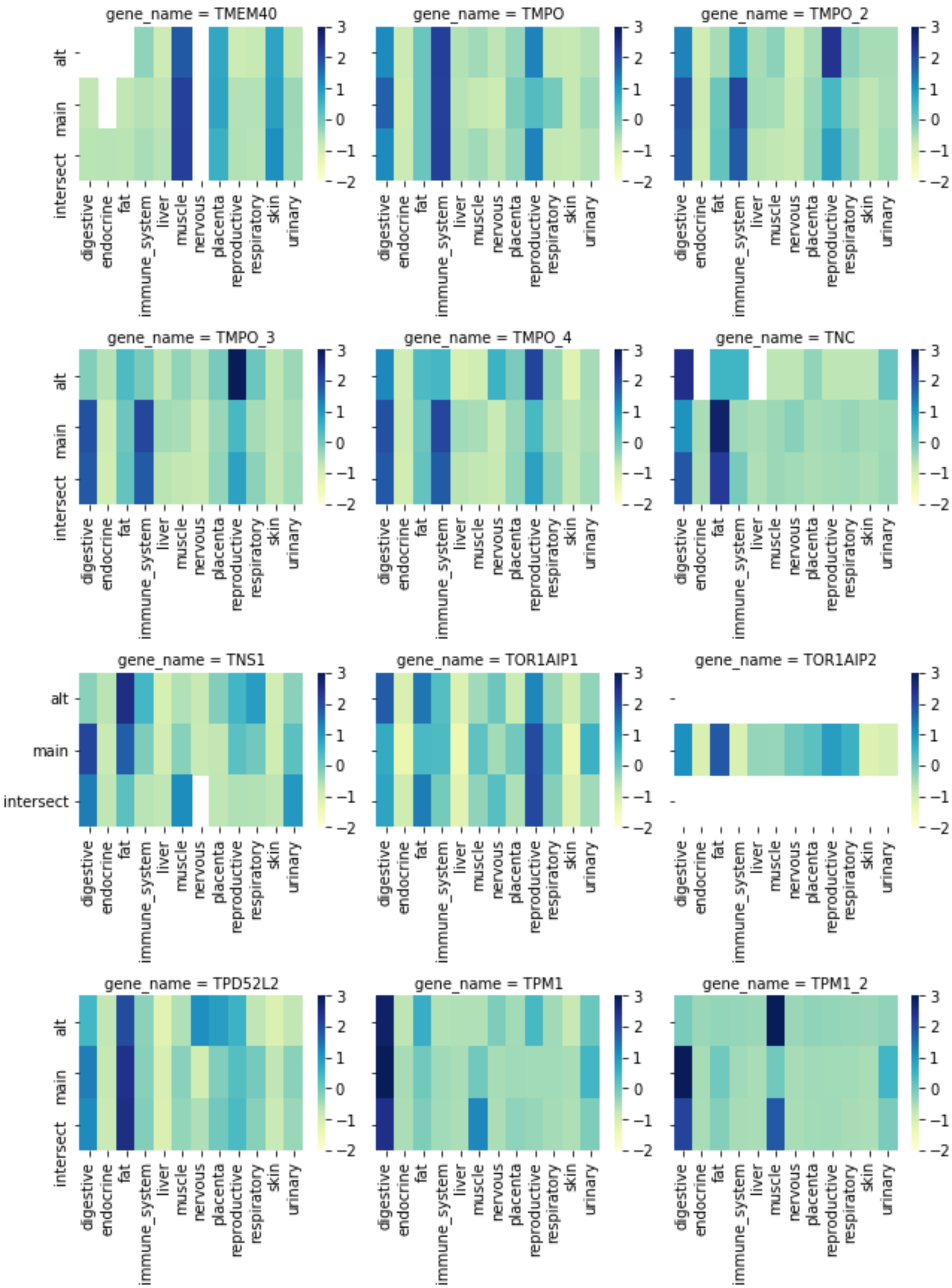

gene_name = TMEM40
gene_name = TMPO
gene_name = TMPO_2
gene_name = TMPO_3
gene_name = TMPO_4
gene_name = TNC
gene_name = TNS1
gene_name = TOR1AIP1
gene_name = TOR1AIP2
gene_name = TPD52L2
gene_name = TPM1
gene_name = TPM1_2
alt
main
intersect
digestive
endocrine
fat
immune_system
liver
muscle
nervous
placenta
reproductive
respiratory
skin
urinary
3
2
1
0
−1
−2

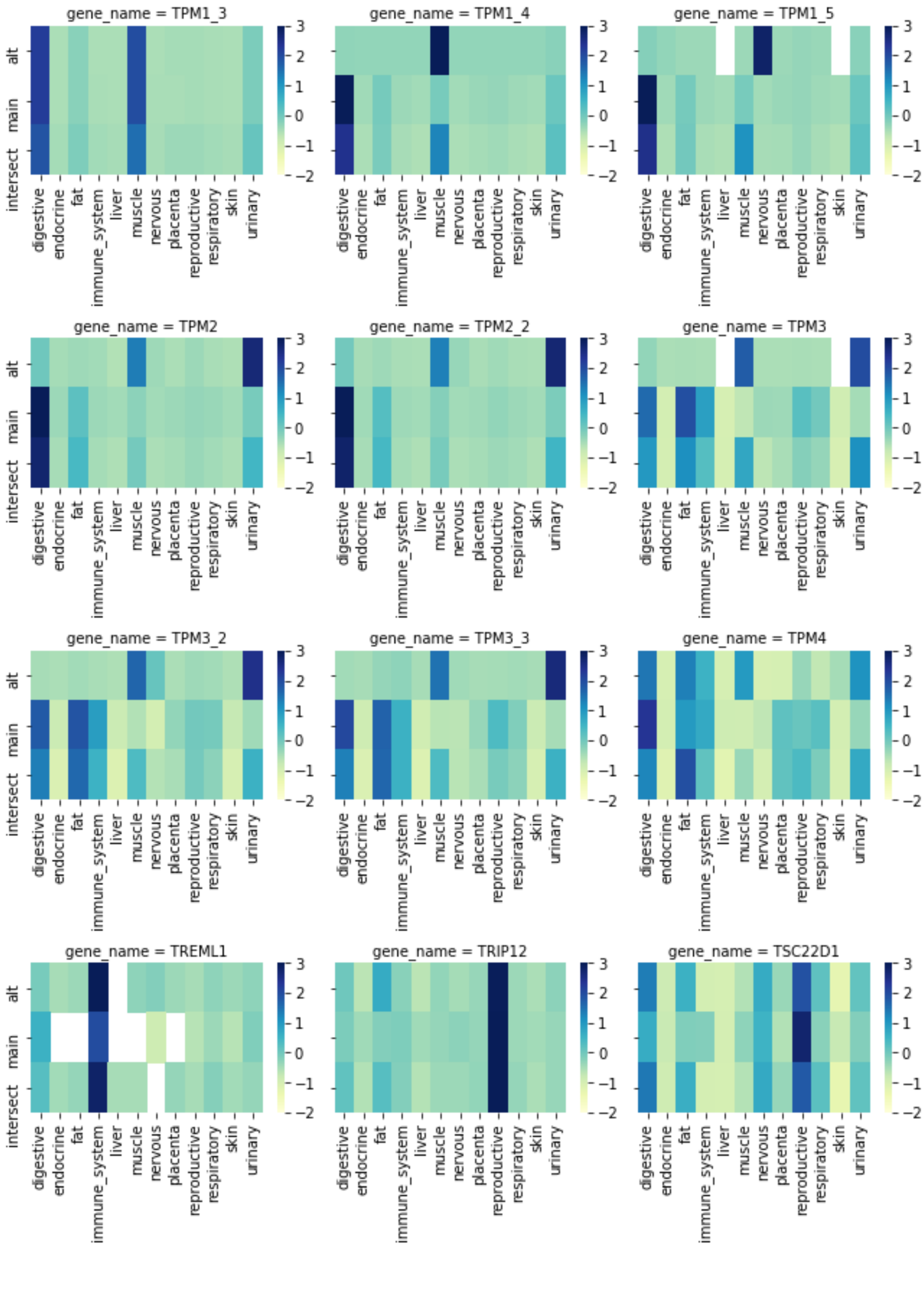
gene_name = TPM1_3
gene_name = TPM1_4
gene_name = TPM1_5
gene_name = TPM2
gene_name = TPM2_2
gene_name = TPM3
gene_name = TPM3_2
gene_name = TPM3_3
gene_name = TPM4
gene_name = TREML1
gene_name = TRIP12
gene_name = TSC22D1
alt
main
intersect
digestive
endocrine
fat
immune_system
liver
muscle
nervous
placenta
reproductive
respiratory
skin
urinary
3
2
1
0
−1
−2

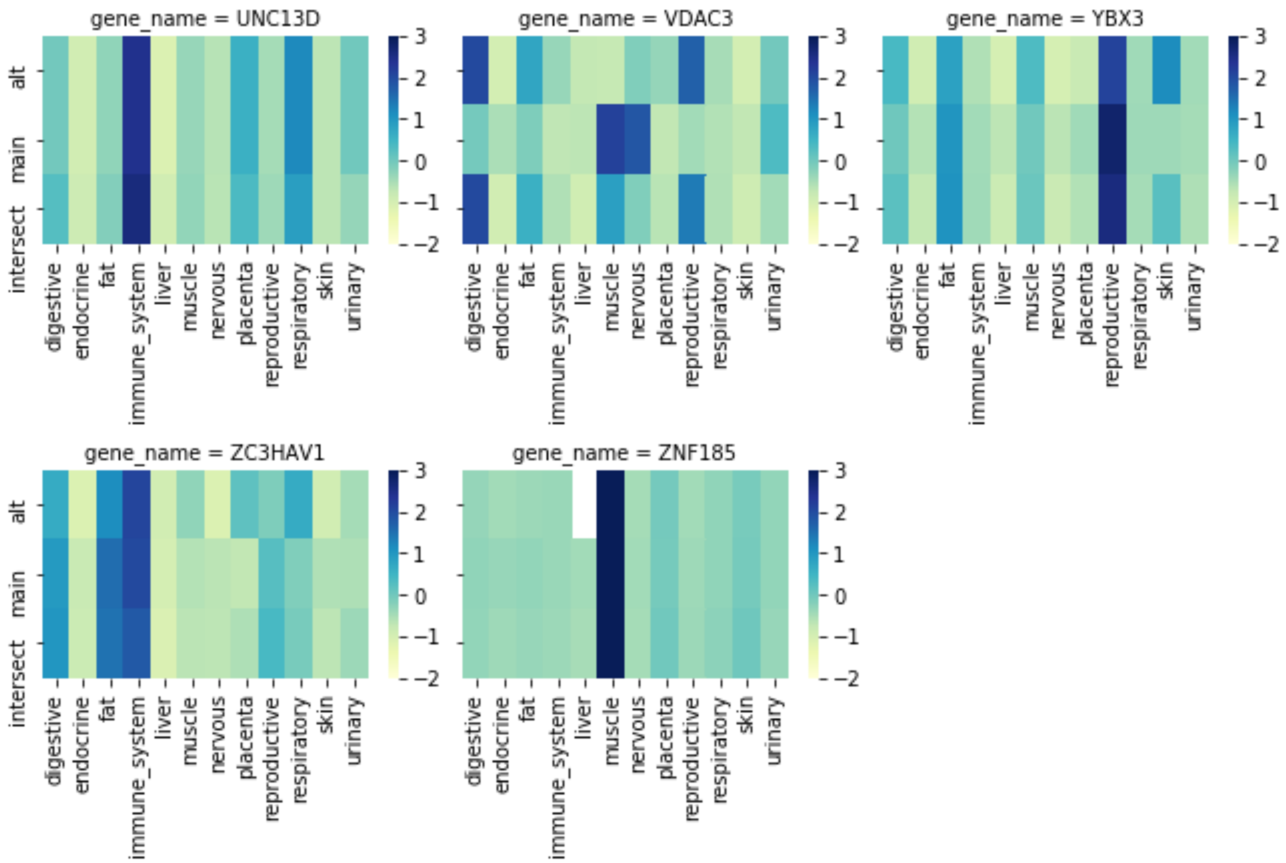

S7 Figure. Heatmaps with the standard deviation for the AS events.

We calculated the mean number of reads across all the tissue groups for each gene and used the mean to calculate standard deviations for each set of reads that mapped to each peptide. Events were counted as tissue group specific when the reads that mapped to one side of the splice event (equivalent to the main protein isoform or the alternative splice isoform) were at least one standard deviation higher than the other side of the splice event.
