# Supplementary material for: An analysis of tissue-specific alternative splicing at the protein level
Source: PLoS Comput Biol. 2020 Oct 5;16(10):e1008287. doi: 10.1371/journal.pcbi.1008287 (PMC7561204; doi:10.1371/journal.pcbi.1008287)
Supplement: Attachment — Submitted filename: Review of Tress et al.docx [file pcbi.1008287.s011.docx]

Review of PCOMPBIOL-D-20-00204

The paper addresses one of most central questions of the eukaryotic biology – what is the role of alternative splicing? Many opinions concerning this topic have been expressed, ranging from ones that alternative splicing is what enabled the evolution of multicellular organism, to ones that it is largely noise. However, there are few rigorous analyses providing evidence one way or another, thus every little step in this field is important. The authors make such a step by reanalysing proteomics data from Pandey lab published in a somewhat controversial paper in 2014, in combination of transcriptomics data from the Human Proteome Atlas.

The bioinformatics analysis is generally done thoroughly, and the manuscript written clearly, however I found a couple of concerns or analysis omissions, which I think can be addressed.

1) What about the change in the overall expression levels of a gene between conditions between which the alternative splice event happen? If the overall gene expression level in one condition is different from that in the other by an order of a magnitude, then the interpretation of the alternative splicing event would be rather different from the cases where in both conditions the gene overall expression level (i.e., summing up all splice forms) is of the same magnitude. The first case may be easily noise. How often each type of events happens? Which are the events of the second category? Making this assessment on proteomics level may be tricky, however at least on RNA level this should be done and added to the analysis.
2) In the Results subsection “Comparison to tissue-specific splicing at the transcript level”, I didn’t find a clear answer to the (in my mind) most important question: what proportion of the tissue specific alternative splicing events observed at the proteomics level have a clear support on RNA level?
3) The authors rightly say that the some of the conclusion of the paper are confounded by the proteomics and RNA datasets are not being paired – the samples analysed are different. Am I wrong to think that in the last few years Human Proteome Atlas have done their own proteomics assays and the proteomics data are available? May be for some reason these data are not appropriate for the analysis that the authors do, however it must be possible to find an additional proteomics dataset for at least two of the tissues. The paper would be much stronger if some of the analysis could be replicated on a second dataset.
4) I would not draw any conclusions regarding the testis tissue: many more genes are expressed in testis on high levels than in other tissues, which will confound any comparisons.
5) The initial part of the Conclusions largely repeats what was already said earlier in the paper. What really are the main conclusions? Possibly it is difficult to draw strong definite conclusions from any individual analysis in this field, but may be the authors should use a part of the Discussion section for a more general discussion. I found their statement that “Instead, our results suggest that much tissue-specific alternative splicing has been involved in the evolution of vertebrate organs, principally heart and brain” important, and perhaps the main specific conslusion. Maybe the authors want to elaborate on this.
6) It has to be noted that the author found only a small number of events, however this fact is not reflected in Abstract. There is even smaller number of events found affecting the “interior” of the gene (9?). Even if these small numbers are in partl caused by the limitations of the available data, it is important to note that “more than the third of events detected” actually correspond to a rather small number overall number. When I was reading the Abstract for the first time, I reached a very different conclusion than after reading the whole manuscript. The conclusion really is that among the small number of alternative splicing events that the authors were able to detect in the proteomics data, a third are tissue specific (or perhaps less, if we account for differences in gene expression, see item 1) above). This is not a criticism, mostly likely the number of functionally relevant alternative splicing events really is small.

I minor comment. I was not able to understand Figure 1B. What do the colours use represent. They all look very similar, why not use the whole range?
