# Supplementary material for: An analysis of tissue-specific alternative splicing at the protein level
Source: PLoS Comput Biol. 2020 Oct 5;16(10):e1008287. doi: 10.1371/journal.pcbi.1008287 (PMC7561204; doi:10.1371/journal.pcbi.1008287)
Supplement: Attachment — Submitted filename: ALT_TS_reply.docx [file pcbi.1008287.s012.docx]

Dear Dr Tress,

Thank you very much for submitting your manuscript "An analysis of tissue-specific alternative splicing at the protein level" for consideration at PLOS Computational Biology.

As with all papers reviewed by the journal, your manuscript was reviewed by members of the editorial board and by several independent reviewers. In light of the reviews (below this email), we would like to invite the resubmission of a significantly-revised version that takes into account the reviewers' comments.

In particular, it is important that you clarify the datasets and the backgrounds used. It is also important to discuss fully any confounding issues and to cite all appropriate references in your introduction and discussion.

We cannot make any decision about publication until we have seen the revised manuscript and your response to the reviewers' comments. Your revised manuscript is also likely to be sent to reviewers for further evaluation.

When you are ready to resubmit, please upload the following:

[1] A letter containing a detailed list of your responses to the review comments and a description of the changes you have made in the manuscript. Please note while forming your response, if your article is accepted, you may have the opportunity to make the peer review history publicly available. The record will include editor decision letters (with reviews) and your responses to reviewer comments. If eligible, we will contact you to opt in or out.

[2] Two versions of the revised manuscript: one with either highlights or tracked changes denoting where the text has been changed; the other a clean version (uploaded as the manuscript file).

Important additional instructions are given below your reviewer comments.

Please prepare and submit your revised manuscript within 60 days. If you anticipate any delay, please let us know the expected resubmission date by replying to this email. Please note that revised manuscripts received after the 60-day due date may require evaluation and peer review similar to newly submitted manuscripts.

Thank you again for your submission. We hope that our editorial process has been constructive so far, and we welcome your feedback at any time. Please don't hesitate to contact us if you have any questions or comments.

Sincerely,

Christine A. Orengo

Associate Editor

PLOS Computational Biology

Arne Elofsson

Deputy Editor

PLOS Computational Biology

***********************

Reviewer's Responses to Questions

**Comments to the Authors: Please note here if the review is uploaded as an attachment.**

Reviewer #1: Rodriguez and colleagues study tissue-specific alternative splicing (AS) at the proteomic level. By re-analyzing the large-scale proteomics dataset from Kim et al, the authors perform one of the first (if not the first) studies evaluating tissue regulation of AS directly at the proteomics level. While the data is a bit limited (255 high-confidence events), it is still of interest and some relatively clear patterns emerged. I would thus support publication of this study if a few issues were addressed:

1) As it could be expected given the history of this topic (the proteomic impact of AS), the Introduction is a bit unbalanced. Many of the cited papers are from the senior author, while certain papers, especially those from the Blencowe lab, are missing (e.g. the discussion on Trends in Biochemical Sciences, the evolutionary studies, various papers on splicing regulation and function, etc.). Given that this is a Results (and not an Opinion) article, it will be important that the authors make an extra effort to be as objective and balanced as possible.

The referee has highlighted a problem with the introduction. At one point in the introduction we went off at a tangent and discussed the concept of alternative splicing and noise. It was not really relevant to this paper, so we have rewritten a couple of paragraphs and lost a couple of references to our work that were in the introduction.

We were a bit surprised to be asked to add more papers from a specific group. We do reference two papers from the Blencowe lab; one because it is highly pertinent to the debate and the other because as far as we know there are no other similar papers. It is possible that we could add references to another paper from the Blencowe group alongside the references we already have, but we already have perfectly good references that have the advantage of being Open Access.

2.1) Overall, it is complex to figure out which are the 255 AS events the authors are studying. A text table with the AS events, with their protein and genomic coordinates, and the peptide counts in each tissue sample should be provided.

There was a table in the supplementary material, with peptide counts, but looking at it again it did not identify the splice events. We have expanded the table so that the events can be identified.

2.2) Also, it would be helpful if the authors also report the events that did not fully met their threshold criteria (at least 3 PEDs for each variant), since AS events with 2 PEDs could also be very interesting.

We could report them, but even if the evidence for these alternative splicing events at the protein level is fairly solid, we would not be able to determine whether they are tissue specific or not.

For these cases, I wonder: if a given tissue (e.g. Adult Frontal cortex) has only two replicates, would all tissue-specific AS events from that tissue be systematically excluded (i.e. those whose alternative isoforms are only detected in that tissue)?

It is clearly a disadvantage to have only two experimental replicates; the fewer replicates there are, the less chance we have of capturing 3 instances. We do specifically go over this in the paper. It is why most of the work concentrates on tissue groups rather than tissues themselves and why the main proteomics-transcriptomics comparisons are between just four of these tissue groups.

If the referee is suggesting that AS events that were specific to one tissue might have been excluded, it is possible, yes. In order to set up the ASE255 set we counted up all the PSMs over all experiments (PEDs) without checking the tissue specificity (TS) of each peptide. So validated splice events might have sufficient PEDs from three different tissues, or from the same three tissues. It wouldn’t matter where the PEDs came from.

If the alternative isoform were highly tissue specific and that tissue only had two replicates, it would certainly be harder to detect enough PEDs for the event to enter the analysis. However, these events would not be automatically excluded, since many AS events can be validated by more than one different peptide, even microindels can be validated by PSMs for the peptide that crosses the indel and the missed cleavage version of the same peptide. In that case it would be theoretically possible to include the event if peptides were detected in different experiments and different peptides were detected in the same experiment.

In any case there were 16 tissues with just two experimental replicates, Adult B-cells, Adult Colon, Adult gall bladder, Adult kidney, Adult monocytes, Adult ovary, Adult pancreas, Adult prostate, Adult rectum. Adult spinal cord, Adult testis, Placenta, Fetal gut, Fetal brain, Fetal ovary and Fetal testis.

As a result of the referee’s question we looked at the data again. There were 81 events for which we detected at least two valid PSM for each side of the event, but that had fewer than the 3 PEDs necessary to calculate statistical significance so were left out of the study. Peptides were detected in replicates of the same tissue for 13 of these events, suggesting that these events could have been tissue specific given enough replicates. The tissues were frontal cortex (4), esophagus (2), fetal heart (2), retina (2), adult heart, pancreas, and fetal liver. Note that all of these tissues actually had more than two replicates. So there were no cases where PSMs were detected in the only two replicates of a tissue. Either these tissues have little AS level specificity, or (more likely) it is a lot harder to spot AS level TS splicing at the protein level if there are only two replicates. We already assumed that it was hard to detect protein level TS in these tissues, which is why we combined results into tissue groups for the study.

In any case, in 7 of these 13 cases we found that both principal and alternative isoforms were expressed in the same tissue, leaving just six cases of possible tissue specific splicing that we could have added to the set (two from frontal cortex, one each for retina, fetal and adult heart, and pancreas) if we had found more PEDs. The events in all six of these genes (*FMN1*, *RAP1GAP*, *NECTIN1*, *IDE3B*, *FRMD5* and *ATP2B3*) are also annotated in fish, showing that all these potentially strongly tissue specific splicing events are ancient too. These results would also fit with what we have already seen in the paper: AS events that are tissue specific at the protein level tend to be highly conserved and enriched in nervous and heart tissue.

This data is briefly described in the caveats section.

This could be artificially reducing the fraction of tissue-specific AS events detected. Therefore, the authors should consider analyzing also those AS events with PEDs in all the replicates of a given tissue (even if these have only 2 PEDs in total). This will expand the small number of studied events (currently 255), which is a weakness of the study.

It is true, 255 events are a weakness. Given the limitations of our study, we know we can’t determine the proportion of splice events that might be tissue specific at the protein level across the whole genome. Despite this, there are still interesting signals in the data. Of course, if we were to double or treble the number of replicates for each study, we would be likely to detect more evidence of tissue specific splicing (but also the number of false positive identifications, which is another story).

Unfortunately, if we add those events that only have 2 PEDs, we will not add anything to the final numbers of TS events. The analysis we carried out above was interesting, but because it is impossible to reach significance with just two PEDs, we will find exactly the same number of significant TS cases.

3) The section "Tissue specificity by event type" and Figure 2 are weak. The categories considered by the authors might be the most functionally relevant from the proteomics perspective, but are difficult to relate to the standard AS event types (cassette exons, mutually exclusive exons, alternative splice site donors/acceptors) and to other forms of transcriptomic variation (e.g. alternative first or last exons by alternative promoters and polyadenylation sites). Figure 2 should include the data split by these event types as well.

We did not carry out this analysis because we could see that the real driving force here was the effect at the protein level. Tissue specificity is related to similarity at the protein level rather than technical aspects of how an mRNA is spliced prior to translation. However, since both referees 1 and 2 asked for it, we would have to assume that most readers may not take our word for it. We added the standard AS event types as a comparison.

Here there are two types of splicing that are enriched in tissue specific events. Almost all MXE events are also homologous exon events. When we removed homologous exon swaps from the Alt Poly A events, the proportion of TS events among the remaining events (the C-terminal class) is actually lower than the proportion of non TS events:

The same thing happens (although to a lesser extent) when the homologous exon swap events are separated from the Alt Prom group (leaving the N-terminal class). Most exon skipping events are indels, most Alt 3’ SS events are micro-indels.

4) The study of the age of the events should also be improved. First, it is unclear which species were searched to define ancient events. Most importantly, the comparison with their genome background does not seem fair. If I understood correctly, they defined as "alternative events" those sequences that do not overlap the principal isoform defined in APPRIS. While this is a good idea, it misses an important group of AS events: those sequences that are generally included, but that are skipped in a (smaller) fraction of transcripts. These AS events will be more ancient according to their definition. The easiest way to assess the impact of this omission is to see for how many of the 255 events the alternative sequence is in the principal isoform. Only those that do not overlap the principal isoform can be fairly compared with their current genome background. Then, ideally, the authors should also come with another background set: sequences in the principal APPRIS isoform that are missing in any of the non-principal isoforms. And determine their evolutionary age, for comparison. Finally, as a minor point, the exclusion of exons smaller than 42 nucleotides is also expected to introduce biases, since small exons have been shown to be particularly conserved and tissue-specific.

The referee makes a good point, we hadn’t included any description of the manual curation. We have now corrected that error. Splice events were not penalised for being short in BLAST searches and we did not limit our searches to BLAST searches either.

With regards to the age of all alternative exons in the genome, we assume that the cases that the referee is describing correspond to cases when the exon is occasionally spliced out of the mature mRNA. Skipping events will produce two transcripts, one with and one without the skipped exon. If the exon is rarely skipped, and more to the point forms part of a conserved feature, APPRIS will generally choose the longer transcript as the most likely to produce the principal isoform. In these cases, the alternative transcript will not contain the skipped exon.

For the 255 cases in our set we could look for the conservation of these deletions because we were evaluating whether both sequences that resulted from the splice event were conserved and there were only 255 events.

In our genome-wide analysis an exon that is occasionally skipped will count as principal and will not have been analysed. This is entirely logical - in many cases the splicing out of the exon will result in damage to a functional domain or structure, or to protein isoform that does not have cross-species conservation. The exon in the alternative transcript may also be skipped in other species. What we would like to measure in the case of the skipped exon is whether the variant without the skipped exon has cross-species evidence. But we can’t do that with a BLAST search, and we have to have some sort of automatic analysis for the whole gene set. For the analysis we had to assume in the first instance that the principal was conserved (it isn’t always) and used BLAST to look for conservation of just the alternative side of the event, which had to be at least 42 bases long.

After the BLAST search we were able to check for conservation by hand.

From a purely observation-based view, we didn’t see any evidence to suggest that these splicing out events are more conserved than any other type of event. Where there might be some sort of bias in the analysis is with the shorter exons. We had to leave exons shorter than 42 bases out, because BLAST would not identify them, but there is some evidence to suggest that shorter exons (those less damaging to any resulting protein) are more likely to be functional than longer exons. Although curiously, in our analysis, the shorter exons in the ASE255 set (micro-indels) were generally less conserved than other exons.

5) Their results suggest that the digestive tissue RNA-seq they analyzed is problematic, and this may be affecting the transcriptomic comparison. For example, the conclusion that "the tissue group with the most evidence for group-specific expression is digestive tissues" is at odds with every previously published study. Hence, I suggest that the authors use other digestive tissue RNA-seq, even if they are from a different source, to perform the transcriptomic vs proteomic comparison.

The referee does make a good point, it is a somewhat unusual result. However, we doubt that there are any substantial problems with the digestive tissues in the Human Protein Atlas RNAseq experiment.

It is much more likely that the differences we see here are related to the data set we are using. There are only 255 events and these events are biased in that they are generally highly conserved, they are all in coding regions (remember practically all RNAseq AS studies are carried out using UTR codons too and often these are the most differentially spliced), and in genes that are highly expressed.

Finally the numbers of digestive TS events are not that much higher than those of fat or nervous or reproductive tissues. Heart tissue specific splicing is lower than might be expected perhaps. Again, all this is likely to be more to do with the idiosyncrasies of our smaller data set than the RNAseq data themselves.

6) I found that the transcriptomic vs proteomic comparison is a bit convoluted and difficult to follow. Could this be simplified in the main text? In the end, their point comes across, but I wonder whether all the details to get the PGE99 and TGE355 sets are really needed in the main text.

The referee is right. Some of the authors struggled to follow the discussions too! First of all, we have removed all reference to TGE355 because we did not really use this set in the end. We have also tried to make it more concise so that it is easier to follow for the reader.

Also, as mentioned above, the result comparing proteomics to transcriptomics is clear, but the opposite is not, likely due to the noisy nature of some of the RNA-seq samples. As suggested above, perhaps by using better transcriptomic samples it will be possible to spare the reader the details of Figure 6, which really have little to do with the rest of the study and does not add much.

We believe that maybe we made this final part of the results section even more opaque, but we believe that it is interesting. In fact these results do more than anything to suggest that there is not much wrong with the digestive samples - the proteomics results essentially agree with the transcriptomics studies in digestive tissues (most points are below the diagonal line), which suggests that many of the events specific at the RNA level in digestive tissues may well have been significantly tissue specific at the protein level, if there had been enough valid discriminating peptides. Since we believe that it is important, we have rewritten this section too. We kept figure 6.

Finally, I wonder: do measures by RNA-seq and mass-spec correlate? E.g. the z-scores across tissues of the ratio of isoforms (or "sides").

This is a very good idea, though we were not sure how to carry out a correlation using the Z-scores from so few peptides. However, we were able to correlate % supporting RNAseq reads and % supporting PEDs across the four tissues. Nervous tissues in particular had a surprising high correlation (0.8). Primate events had very little correlation at all.

From left to right, top to bottom: digestive (blue), nervous (yellow), muscle (red) and reproductive (green)

7) A formal point: I found the concept "side of the splicing event" is a bit mis-leading. What does "side" refer to here? Is there a better way to refer to this? What about simply "variant"? The first time I read I thought this was referring to the two sides of an exon (upstream and downstream exon junctions).

The difficulty is that we can only analyse the use of splicing events since neither RNAseq reads or peptides are long enough to cover whole transcripts or isoforms. If we could look at transcripts and proteins it would be much easier to describe the results. If they were all of one type (as in many transcript level studies where they concentrate only on skipped exons) there might also be a way of describing (spliced in etc). But our data is a mixture of different types of splicing events. We can’t see a way around using the sides of the events to describe mapping events.

It is true that we never defined the sides of the event properly, so now we have defined it in the methods section.

Reviewer #2: I found this work on the whole interesting and well put together. The functional role of alternative splicing is controversial and I think this work helps to further identify a subset of minor isoform's that everyone can agree have promising functional signatures. Thanks to the authors for providing this interesting analysis that I enjoyed reading.

Requested Revisions

You need to detail and then justify the background sets you used for the DAVID analysis. I think in this case its just a matter of being more explicit. It could be that you used the whole genome for the background. If this has been done, then I don't think this would be appropriate background. So some clarification on the backgrounds is needed.

We had carried out the analysis against the whole genome. In the end it made sense to use as background those 10,485 genes for which we detected at least two peptides, since it was only possible to detect alternative splice variants for these genes.

With regards to the accessibility of the paper, its good to keep in mind that many people reading the paper will not be quite as familiar with

the definitions. In Figure2 B the different classes could have expanded explanations as to how they are precisely defined to make the results more accessible (in a supplementary table). For example can C-terminal N-terminal potentially come from variable promoter usage. That's not a problem if they do, but further detailed qualifications of what types of events could belong to each group would be helpful in a table.

Two referees have asked for this type of graphic. As explained above we believe that the signal is most clear at the protein level, but we have carried out the analysis because it is an interesting comparison. We show it alongside the protein level splice events.

I suspect there could be some confounding factors that aren't fully addressed. For example, short indels are less tissue specific but also presumably very difficult to detect through homology searches (so will rarely be identified as conserved even if they are) .

This would then make tissue specific events, enriched in homologous exons, appear to be more ancient because the homologous exons are so much easier to detect using sequence searches.

I may have interpreted this wrong but I would be interested to hear from the authors about this. If there are genuine caveats here perhaps some extra text on this could be warranted in the caveats section.

The problem here is that we did not describe how we carried out the manual curation of event ages in the paper (as referee 1 pointed out too) so that has now been added.

It is true that BLAST will struggle to identify short similar sequences. However, we did not rely solely on BLAST hits for manual event age identification. In addition, when we did carry out BLAST searches against distant species, we extended short exons (and deletions, of course!) by up to ten amino acids in each direction. We also carried out multiple alignments of the whole sequence search for conservation to other species. We required that both sides of the event were conserved in the most distant species possible.

In the case of the homologous exons we were not content with just finding homology in a distant species. For the homologous exon events we required both exons to have homology to different homologous exons in two different transcripts in the correct part of the sequence and that each homologous exon was clearly orthologous to one of the two two exons and not the other. The homologous exon events really are all conserved in fish and some of them are conserved across vertebrates too (but that’s another story).

Suggested

Missing full stop after this sentence "but most were generated by NAGNAG splicing [38]" also perhaps have a sentence defining NAG NAG splicing e.g. but most were generated by NAGNAG splicing [38] a form of splicing where....

Done.

Figure4 B the color charts are difficult to follow because they are all shades of maroon. You may want to think about making the color bars more distinguishable for different tissues.

More than one referee has complained about this, we have found a better colour system to replace this.

I didn't quite follow the logic of this sentence "If conserved post-translational

buffering systems exist, this would remove the need for transcript level constraints." I think expanding the sentence to spell out the meaning would be helpful.

We have left this out of the introduction.

Figure 1 seems quite stranded in the methods section and is only referred to as figure 1C from the text. Usually its better if all aspects of a figure are referred to from the text. Otherwise it suggests the figure needs reworking. I think an example here is good, but it needs work to integrate it into the text more.

The referee is quite right. We have integrated it into the text along with a description required by referee 1. We have also changed the colour system ...

In the Author summary I felt this sentence needed improving:

"and the three quarters of primate-derived alternative exons may not behave the same way."

The referee is right, it was somewhat confusing. It now says “Just 5% of annotated alternative exons in the human genome are ancient while three quarters of alternative exons are primate-derived. None of the primate-derived events in our analysis were tissue specific at the protein level.”

In the following section it may be important to bring in the idea and contribution of other processes such as alternative promoter usage.

"This is reflected in the human reference set; at present human coding genes are

annotated with an average of four distinct gene products [3]. Recent studies suggest that human coding

genes generate on average more than ten alternative transcripts [4, 5]"

The opening sentence of the introduction now says “Almost all multi-exon genes are able to undergo alternative splicing [**1, 2**] via a range of mechanisms which include exon skipping, alternative splice site usage and alternative promoter and poly-A usage.”

Replace Gtex with GTEx

Done

What looks like a minor omission in the analysis is how this work closely relates to

<https://www.ncbi.nlm.nih.gov/pmc/articles/PMC3437557/>

Not that you would have to do any analysis, but you could have something in the conclusions, since one question for me that's left open is what are the alterations in protein structure for the tissue specific events relative to the Babu paper.

This is a very interesting question. We don’t have time to analyse everything related to the Babu paper, but we did have a look at disorder using the same predictor and the same cut-offs as Babu. We find that the alternative events were enriched in disorder, but the tissue specific events had relatively fewer disordered events, which disagrees somewhat with their paper. This was also true even when we analysed just the cassette exons (like the Babu paper). Disordered regions were most enriched in more recent splicing events.

We believe that much tissue specific alternative splicing will affect protein-protein interactions in distinct tissues, but our study suggests that the role of disorder may not be as important as has been suggested. The analysis should come with the proviso that disorder predictors in general have a relatively high false positive rate. Events in tropomyosin were predicted as disordered, but its 3D structure has been solved and it has no disorder whatsoever.

General comments

I found the text on the whole very readable. However, the section "Event age and tissue groups" was difficult to wade through. I would suggest distilling this section down a bit, because as it stands some readers may struggle and not pick out the key results and their implications.

We have taken up the referee’s suggestion. We hope that it is more readable now.

I liked the way that you put some emphasis on the need to functionally characterize a subset of splicing events that look functional. With the advent of CRISPR technologies experiments the ability to investigate these functions is becoming far easier. Given your groups expertise I wonder if the paper from this data could be combined with other data in APPRIS to provide a webpage formatted as a data table entitled "subset of minor isoforms recommended for functional characterisation" . Tissue specific proteomic detected isoforms could be one way of adding entries to this table. This is just a suggestion and would require some work and isn't necessary for publication. But I think would add to the paper if the current submission is not successful.

We would like to thank the referee for this excellent idea. We will add a web page with some of the tissue specific cases right away. In fact we are doing further work in this area, so we can add to the list as we make further advances in this field. The web page is available on the APPRIS web site now (the “Important Isoforms” section in the top menu).

Reviewer #3: The paper addresses one of most central questions of the eukaryotic biology – what is the role of alternative splicing? Many opinions concerning this topic have been expressed, ranging from ones that alternative splicing is what enabled the evolution of multicellular organism, to ones that it is largely noise. However, there are few rigorous analyses providing evidence one way or another, thus every little step in this field is important. The authors make such a step by reanalysing proteomics data from Pandey lab published in a somewhat controversial paper in 2014, in combination of transcriptomics data from the Human Proteome Atlas.

The bioinformatics analysis is generally done thoroughly, and the manuscript written clearly, however I found a couple of concerns or analysis omissions, which I think can be addressed.

1)	What about the change in the overall expression levels of a gene between conditions between which the alternative splice event happen? If the overall gene expression level in one condition is different from that in the other by an order of a magnitude, then the interpretation of the alternative splicing event would be rather different from the cases where in both conditions the gene overall expression level (i.e., summing up all splice forms) is of the same magnitude. The first case may be easily noise. How often each type of events happens? Which are the events of the second category? Making this assessment on proteomics level may be tricky, however at least on RNA level this should be done and added to the analysis.

In proteomics if this happens (gene expression drops off dramatically in one tissue) it is likely that you won’t record any/enough peptides in that tissue to make a comparison possible. For example gene *ACSL6* is practically entirely detected in reproductive and nervous tissues in the proteomics experiments, though we do detect the odd peptide for the endocrine and digestive tissues. If one of the isoforms was specific for one of these tissues, there would not be enough PEDs to determine whether it was specific or not. We can’t detect tissue specificity in these cases. It is one of the limitations of the proteomics data, but at least we know we aren’t seeing noise.

However, we looked in detail at our transcriptomics data again and we found that the referee was right. First there were two genes that had so little transcription evidence for one side of the event that it made the comparison inviable (*TNC* and *MPIG6B*). These two genes were removed entirely from the transcript level analysis. Neither case was significantly tissue specific at the protein level. In addition, the evidence in certain tissues in three other genes was not sufficient to support RNAseq level tissue specificity on one side of the event, and so these three events were no longer counted as tissue specific at the transcript level in these tissue groups.

The numbers of events tissue specific at the transcript level have changed slightly as a result. This did not affect the protein level results.

2)	In the Results subsection “Comparison to tissue-specific splicing at the transcript level”, I didn’t find a clear answer to the (in my mind) most important question: what proportion of the tissue specific alternative splicing events observed at the proteomics level have a clear support on RNA level?

We did include this number in the paper, but the wording was a bit strange. Now it says: “Reassuringly, we found that two thirds (66) of the protein level enrichments were enriched in the same tissue group at the transcript level.”

3)	The authors rightly say that the some of the conclusion of the paper are confounded by the proteomics and RNA datasets are not being paired – the samples analysed are different. Am I wrong to think that in the last few years Human Proteome Atlas have done their own proteomics assays and the proteomics data are available? May be for some reason these data are not appropriate for the analysis that the authors do, however it must be possible to find an additional proteomics dataset for at least two of the tissues. The paper would be much stronger if some of the analysis could be replicated on a second dataset.

No, the referee is quite right. We have also seen the data. Unfortunately the data came out when we had already carried out the analysis (our paper has been with the referees 5 months now due to complications with the Coronavirus, we assume).

We would love to redo the analysis with the Wang data, but we would have to change the whole analysis to do it largely because the Wang analysis does not include replicates in the same way as the Kim analysis.

4)	I would not draw any conclusions regarding the testis tissue: many more genes are expressed in testis on high levels than in other tissues, which will confound any comparisons.

We have now made it clear that any conclusions we make about testis data are tentative given the nature of the tissue.

5)	The initial part of the Conclusions largely repeats what was already said earlier in the paper. What really are the main conclusions? Possibly it is difficult to draw strong definite conclusions from any individual analysis in this field, but may be the authors should use a part of the Discussion section for a more general discussion. I found their statement that “Instead, our results suggest that much tissue-specific alternative splicing has been involved in the evolution of vertebrate organs, principally heart and brain” important, and perhaps the main specific conclusion. Maybe the authors want to elaborate on this.

The referee is right about this too. The conclusions, especially the first part, needed to be more concise. We have now rewritten the conclusions. We hope they have improved.

6)	It has to be noted that the author found only a small number of events, however this fact is not reflected in Abstract. There is even smaller number of events found affecting the “interior” of the gene (9?). Even if these small numbers are in partly caused by the limitations of the available data, it is important to note that “more than the third of events detected” actually correspond to a rather small number overall number.

The phrase “more than a third of the alternative events we detected” in the abstract refers to a third of the whole set of 255 events. We are not sure how to answer the rest of the question because we do not use the word “interior” anywhere in the article, nor do we have any data sets as small as 9 in the article.

When I was reading the Abstract for the first time, I reached a very different conclusion than after reading the whole manuscript. The conclusion really is that among the small number of alternative splicing events that the authors were able to detect in the proteomics data, a third are tissue specific (or perhaps less, if we account for differences in gene expression, see item 1) above). This is not a criticism, mostly likely the number of functionally relevant alternative splicing events really is small.

The referee is quite right here. We have made wholesale changes to the abstract to reflect this better.

I minor comment. I was not able to understand Figure 1B. What do the colours use represent. They all look very similar, why not use the whole range?

We had the same problem with figure 4 for a different referee. We have updated both this figure and figure 4 to include a larger range of colours.
